# LC-MS/MS Method Development for the Discovery and Identification of Amphidinols Produced by *Amphidinium*

**DOI:** 10.3390/md18100497

**Published:** 2020-09-29

**Authors:** Marvin Wellkamp, Francisco García-Camacho, Lorena M. Durán-Riveroll, Jan Tebben, Urban Tillmann, Bernd Krock

**Affiliations:** 1 Alfred-Wegener-Institut für Polar und Meeresforschung, 27570 Bremerhaven, Germany; marvin.wellkamp@web.de; 2 Chemical Engineering Department, University of Almería, La Cañada, 04120 Almeria, Spain; fgarcia@ual.es; 3 CONACyT-Departamento de Biotecnología Marina, Centro de Investigación Científica y de Educación Superior de Ensenada, Ensenada 22860, Mexico; lduran@conacyt.mx; 4 Section Ecological Chemistry, Alfred Wegener Institute, Helmholtz Centre for Polar and Marine Research, 27570 Bremerhaven, Germany; jan.tebben@awi.de (J.T.); urban.tillmann@awi.de (U.T.)

**Keywords:** phycotoxin, ichthyotoxin, dinoflagellate, harmful algae, glycosylation

## Abstract

Amphidinols are polyketides produced by dinoflagellates suspected of causing fish kills. Here, we demonstrate a liquid chromatography-tandem mass spectrometry (LC-MS/MS) method for the identification and quantification of amphidinols (AM). Novel AM were detected by neutral loss (NL) scan and then quantified together with known AM by selection reaction monitoring (SRM). With the new method, AM were detected in four of eight analyzed strains with a maximum of 3680 fg toxin content per cell. In total, sixteen novel AM were detected by NL scan and characterized via their fragmentation patterns. Of these, two substances are glycosylated forms. This is the first detection of glycosylated AM.

## 1. Introduction

Amphidinols (AM) are a group of secondary metabolites produced by dinoflagellates of the genus *Amphidinium*. This genus of predominantly benthic species encompasses a number of naked and dorsoventrally flatted species of temperate and tropical waters [1]. Phototrophic *Amphidinium* species are important contributors to primary productivity in the interstitial zone and form abundant blooms that cause sand discoloration [2]. *Amphidinium* blooms have also been suspected to be associated with fish kills [3]. Based on morphological and phylogenetical evidence, the genus was redefined based on rather stringent morphological criteria as species with a minute, crescent-shaped or triangular epicone [4]. This “*Amphidinium* sensu strictu” now includes ca. 20 of the originally 120 species of the old genus, and many of the other species have been reclassified or are listed as “*Amphidinium* sensu lato” [4,5]. The first AM was reported in 1991 from the cultures of *Amphidinium klebsii* in Japan [6]. The hairpin-shaped polyketides are characterized by a core unit, consisting of two tetrahydropyran rings which are separated by a C_6_ chain, a polyunsaturated (lipophilic) alkyl arm, and a long polyhydroxy (hydrophilic) arm [7]. There are several subgroups of the AM family that share the same core unit but differ in length and structure of the lipophilic and hydrophilic arm, namely amphidinols (AM), luteophanols (LP), lingshuiols (LS), symbiopolyols (SP), karatungiols (KAR), and carteraols (CAR). In contrast, amdigenols (AMD), which also belong to the long-chain polyketides, occupy a special position since the extremely large molecules AMD-A and AMD-E are composed of two AM core units, which leads to the assumption that they are dimers of AM. AMD-G, on the other hand, shares the typical AM structure with one core unit [8]. The different names of otherwise closely related substances result from the lack of rules for the assignment of trivial names to chemical compounds. The rules of systematic nomenclature of chemical compounds established by the International Union of Pure and Applied Chemistry (IUPAC) would result in very long and unhandy chemical names for such big molecules as AM. For this reason, trivial names for large polyketides are commonly used, which only depend on the preference of the researcher describing them, often resulting in inconsistencies. For example, the term “luteophanol” was introduced to differentiate LP-A from other AM by its terminal sulfate group and several hydroxyl groups on the lipophilic arm [9]. In contrast, substances discovered later with the same characteristics were assigned to AM (e.g., AM-17 [10] or AM-20 [11]). To avoid confusion, in this work the term “AM” includes all compound groups listed above. The majority of AM have been tested for a variety of biological activities, with antifungal and hemolytic effects observed in almost all investigated AM, i.e., [12,13,14]. In less frequent cases, cytotoxic and anticancer effects were also found [15,16]. Furthermore, a gill-damaging and therefore fish-poisonous or ichthyotoxic effect has been found in a not structurally elucidated substance, which has a retention time and UV spectrum similar to LP-A [3]. The structurally closely-related karlotoxins, produced by species of *Karlodinium*, have also been shown to have an ichthyotoxic effect [17]. *Karlodinium* appears to use the toxins for prey capture [18], with protection against predators being an additional benefit [19]. The toxins create pores in membranes, which increase permeability and, consequently, lead to depolarization, motor dysfunction, osmotic swelling of the cell, and finally to lysis [17]. Further investigations have shown that the hairpin structure and especially the lipophilic polyene part are responsible for the allelochemical effects. For example, AM-7 differs from AM-14 only by two hydroxyl groups on the polyene part of the molecule but has far stronger hemolytic and antifungal activity [20]. Cholesterol-dependent membrane permeabilization properties were also found in several AM analogs [21], but the ecological role and benefit of AM has not yet been identified. However, due to the similar chemical structure to the karlotoxins, a similar function cannot be eliminated.

The ability to detect a wide array of structural AM analogs is a prerequisite to investigate the biological variability and allelochemical roles of this toxin group and to better understand its effects on humans and the environment. This work aimed to develop a standardized and sensitive analytical method for the quantification of AM as well as detection of novel AM. Similar methods have been successfully established for many shellfish toxins and, more recently, for karlotoxins [22]. To achieve the methodological development for AM, we used a purified standard of luteophanol D (LP-D) [11] to establish the method for the LC-MS/MS identification and quantification and then applied this method to other known members of the AM family. Furthermore, we then used neutral loss scans tailored to the fragmentation of known AM to detect novel AM. Finally, the developed methods were tested on several available strains of *Amphidinium*.

## 2. Results

### 2.1. Adduct Formation of Amphidinols

**Figure 1:**
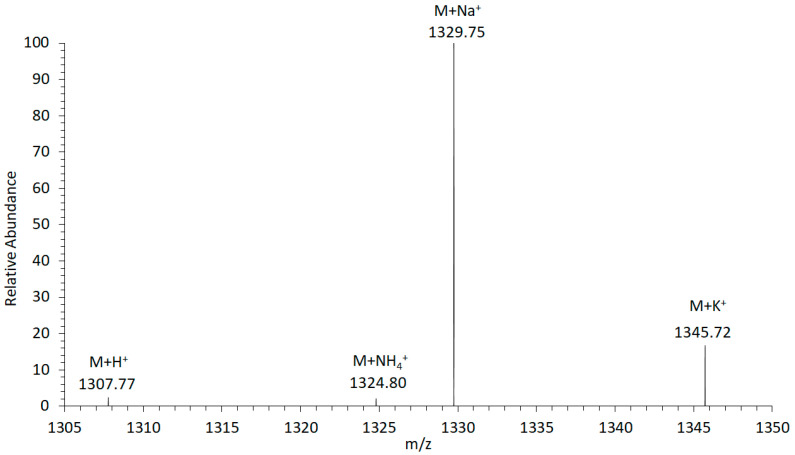
Adduct formation of luteophanol D (LP-D) in electrospray ionization (ESI). The highest ion yield was recorded for the sodium adduct (M + Na^+^).

For the investigation of the adduct formation of amphidinols (AM) under electrospray ionization (ESI) conditions in the ion source of the mass spectrometer, the LP-D standard was tested in the Selected Ion Recording (SIR) mode. The mass range of the most common adducts (H^+^, NH_4_^+^, Na^+^, and K^+^) were scanned. The experiment revealed that the sodium adduct was clearly formed with the highest relative abundance (Figure 1). Accordingly, the sodium adduct was selected as the ionized species for all other AM for subsequent calculations, investigations, and measurements to achieve maximum sensitivity in the detection of this toxin class.

### 2.2. Fragmentation Patterns of Amphidinols

**Figure 2:**
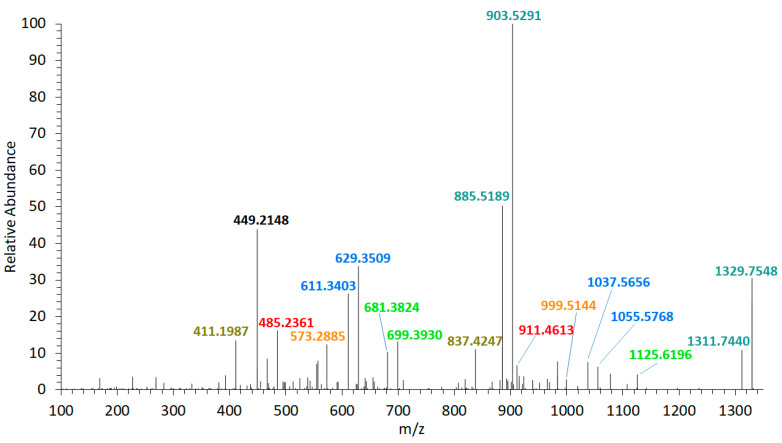
High resolution mass spectrometric (HRMS) collision-induced dissociation (CID) spectrum of LP-D, t_R_ = 2.00 min. Fragment pairs with a difference of 426 Da are marked in corresponding colors. t_R_ = retention time.

**Figure 3:**
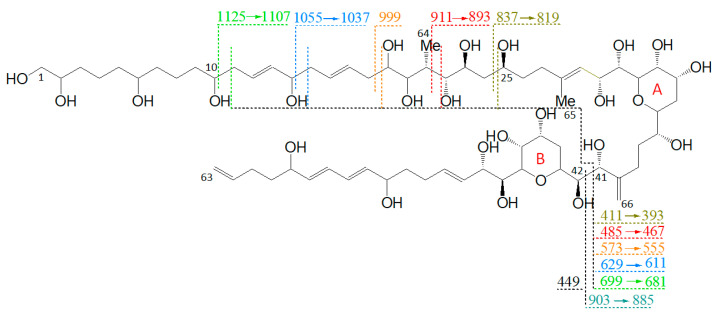
Fragmentation pattern of LP-D. Fragments, which are formed by cleavages in the hydrophilic arm (C10–C25), undergo a second cleavage at the C41–C42 cleavage site, resulting in fragment pairs marked in corresponding colors.

The collision-induced dissociation (CID) spectrum of the sodium adduct of LP-D was characterized by a base peak (100% relative abundance) at *m*/*z* 903.5 followed by a water loss (18 Da) that also was apparent in most of the other fragments and the pseudo-molecular ion (Figure 2). High resolution mass spectrometric (HRMS) analysis (Table 1) revealed that the fragment *m*/*z* 903.5 was formed by the cleavage of the bond C41–C42 in the α-position of the tetrahydropyran ring B (Figure 3).

Furthermore, the fragmentation pattern and HRMS analysis evidenced that all fragments between the pseudo-molecular ion *m*/*z* 1125.6 and *m*/*z* 800, with exception of *m*/*z* 903.5/885.5, resulted from cleavages between C10 and C25. On the other hand, the fragments in the mass range between *m*/*z* 903.5 and 411, except for *m*/*z* 837.4 (which was the result of the cleavage between C24 and C25) and *m*/*z* 449.2, also resulted from the cleavage between C41 and C42. Accordingly, this fragmentation pattern of LP-D resulted in fragment pairs: one fragment with and one without cleavage between C41/42. These fragment pairs were labeled with corresponding colors in Figure 3.

The C41/42 cleavage included the loss of the lipophilic arm (C42–C63) of the molecule and for this reason, the mass difference within all fragment pairs was 426 Da, the molecular weight of the lipophilic arm. Interestingly, there was only one fragment that did not fit into this pattern, which was *m*/*z* 449.2. Even though *m*/*z* 449.2 also was formed by the C41/42 cleavage, it carried the charge and was detected as a fragment in the CID spectrum and not indirectly seen as a neutral loss (NL) as in all other cases.

**Figure 4:**
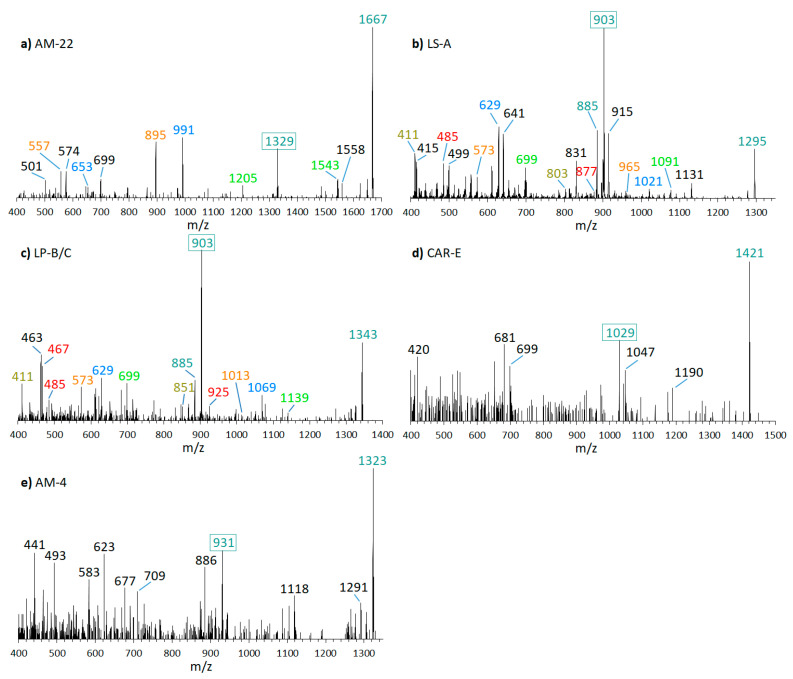
CID-Spectra of amphidinols (AM) were detected in this study. The primary fragment occurring in all AM resulting from the C1/C1′ cleavage at the α-position of the tetrahydropyran ring B is framed, other fragment pairs are shown in corresponding colors. (**a**) AM-22, t_R_ = 2.60 min, strain DN241EHU and CCMP1314; (**b**) LS-A, t_R_ = 2.58 min, strain ACRN02; (**c**) LP-B/C, t_R_ = 2.23 min, strain ACRN02; (**d**) putatively CAR-E, t_R_ = 2.52 min, strain ACRN02; (**e**) putatively AM-4, t_R_ = 2.62 min, strain ACRN02. t_R_ = retention time.

This result prompted a screen of the available strains by a preliminarily selected reaction monitoring (SRM) scan, established on the assumption that all AM sodium adducts form a cleavage at the C-C bond in the α-position of the tetrahydropyran ring B (see Section 2.6 for more information about the SRM scan). In this analysis, several known AM could be detected in different strains. CID spectra of these AM (AM-22, *m*/*z* 1667; LS-A, *m*/*z* 1295; LP-B/C, *m*/*z* 1343; CAR-E, *m*/*z* 1421; AM-4, *m*/*z* 1323; shown in Figure 4) confirmed the fragmentation pattern worked out for LP-D, even though some spectra were not well resolved due to low abundance of the substance. Sometimes not visible at the first glance, all these spectra contained a fragment that was formed by the cleavage of the C–C bond in α-position of the tetrahydropyran ring B (C41–C42), which in the case of LP-D resulted in the high abundant fragment *m*/*z* 903 (Figure 3). In the spectra of AM-22, LS-A, and LP-B/C, several fragment pairs were also visible, even if the heavier fragments resulted from cleavages within the hydrophilic arm only appeared in low abundances. As there were structural variations between the lipophilic arms of the above-mentioned AM, the mass differences between the ion pairs of each AM also varied: 338 Da for AM-22, 392 Da for LS-A, CAR-E, and AM-4, and 440 Da for LP-B/C. Further information on spectra and the corresponding fragmentation patterns for the mentioned AM are shown in Supplementary Materials Figures S1–S6.

As there was strong evidence that the C41–C42 cleavage of LP-D was the central fragmentation site not only of LP-D but of many—if not all—AM, it could be used as a characteristic feature in the interpretation of the CID spectra of AM as it facilitated the recognition of AM and also provided information about the structure of the lipophilic arm, which was of high relevance for AM analysis. However, as in the literature, the carbon atoms are always numbered in ascending order, from the end of the hydrophilic arm to the end of the lipophilic arm, and the arms may vary in their lengths, the preferred and conserved cleavage site of AM resulted in different carbon numbers among different AM and thus cannot be described uniformly for all AM. For this reason, a new carbon numbering was proposed starting at both sides of the main cleavage site: numbers C1 to Cn for the hydrophilic part of AM and C1′ to Cn’ for the lipophilic part. In the following, this modified C-numbering scheme will be used.

### 2.3. Deviating Fragmentation Patterns

**Figure 5:**
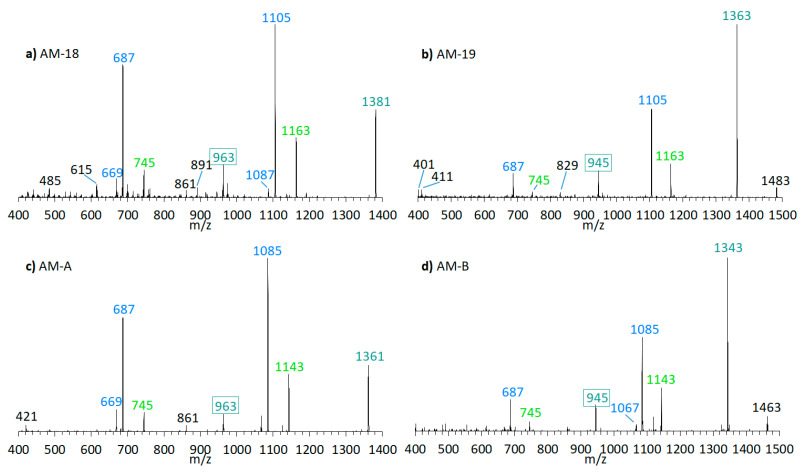
CID-Spectra of detected AM, which only display low abundant fragments resulting from the C1′/C1 cleavage. (**a**) AM-18, t_R_ = 2.71 min, strain CCMP1314; (**b**) AM-19, t_R_ = 2.20 min, strain CCMP1314; (**c**) AM-A, t_R_ = 2.76 min, strain DN241EHU (**d**) AM-B, t_R_ = 2.27 min, strain DN241EHU. t_R_ = retention time.

The preliminary SRM scan also revealed some other known AM, which have been identified by mass spectrometry by Nuzzo et al. (2014) [7] and Cutignano et al. (2017) [23] before. Their CID spectra differ significantly from the AM detected above. In contrast to the fragmentation of LP-D and other AM, the CID spectra of AM-18 and AM-A and their sulfated derivatives AM-19 and AM-B, only displayed low abundant fragments resulting from the C1′/C1 cleavage, which is *m*/*z* 963 for AM-18 and AM-A, and *m*/*z* 945 for AM-19 and AM-B. Instead, they showed additional high abundant peaks that distinguish their spectra strongly from those of other AM, e.g., *m*/*z* 1163, *m*/*z* 1105, and *m*/*z* 687 (Figure 5a).

**Figure 6:**
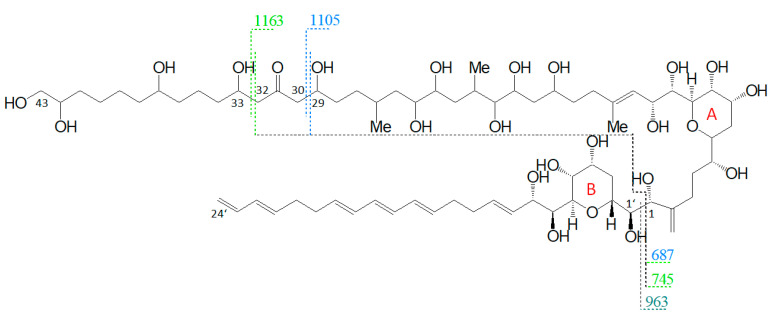
Fragmentation scheme of AM-18, a representative for AM-19, AM-A, and AM-B. The fragments *m*/*z* 687 and *m*/*z* 745 show a difference of *m*/*z* 418 to the fragments *m*/*z* 1105 and *m*/*z* 1163.

Pair fragments were also clearly visible in these spectra. The high abundant fragments *m*/*z* 1163 and *m*/*z* 1105 for AM-18/AM-19 and *m*/*z* 1143 and *m*/*z* 1085 for AM-A/AM-B resulted from cleavage sites in both β-positions of the carbonyl group at C31 which is flanked by a β-hydroxyl group on each side (C29 and C33). AM-18 and AM-19 shared the same lipophilic arm, resulted in a difference between the ion pairs of 418 Da, while the difference between the ion pairs of AM-A and AM-B was 398 Da. Figure 6 shows the fragmentation pattern and the modified C-numbering of AM-18, which is representative for AM carrying a carbonyl group that is flanked by the β-hydroxyl group on each side.

### 2.4. Neutral Loss (NL) Method for the Detection of Unknown Amphidinols

To be able to detect unknown AM variants, an LC-MS/MS method in neutral loss (NL) mode was developed on the base of the molecular weight of all known lipophilic arms of AM from published CID or NMR data that are eliminated during fragmentation as neutral particles by the C1/C1′ cleavage. A total of seven different variants of the lipophilic arm occurred in the 37 currently known AM. To account also for sulfated AM, a second NL method was set up with an addition of 120 Da (Table 2). 

### 2.5. New Amphidinol Candidates

**Figure 7:**
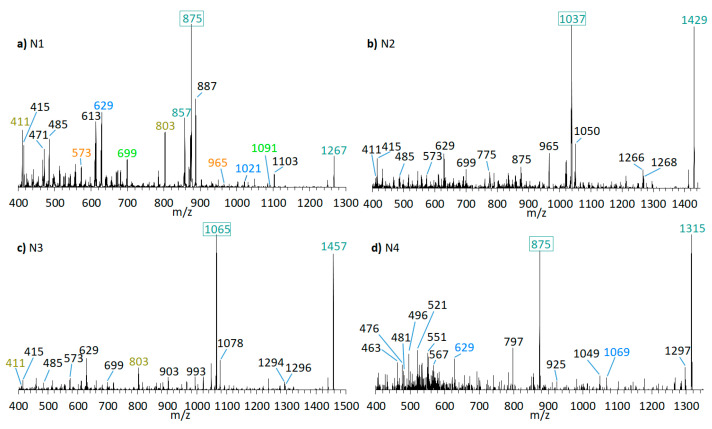
CID spectra of new compounds found in *A. carterae* strain ACRN02. (**a**) N1, t_R_ = 2.56 min; (**b**) N2, t_R_ = 2.52 min; (**c**) N3, t_R_ = 2.53 min; (**d**) N4, t_R_ = 2.16 min. t_R_ = retention time.

Neutral loss experiments of the typical AM losses of 392, 426, 418, 398, 440, 442, and 338 Da, and their sulfated types (Table 2), were conducted on the *Amphidinium carterae* strains ACRN02, ACRN03, CCMP1314, DN241EHU, A01BR and on the *A. massartii* strain AA39, *A. operculatum* strain AA60, and *Amphidinium* sp. strain AA40. Four candidates (N1–N4) for unknown AM were found in the *A. carterae* strain ACRN02 (Figure 7). They correspond to the LP-D type spectra with one prominent fragment resulting from the C1’/C1 cleavage. The substance N1 (*m*/*z* 1267, (Figure 7a) was 28 Da lower than LS-A (*m*/*z* 1295), which was also detected in strain ACRN02 (Figure 4b). N2 (*m*/*z* 1429, Figure 7b) and N3 (*m*/*z* 1457, Figure 7c) were 162 Da higher than N1 and LS-A. High-resolution mass spectrometry (HRMS) has shown that the NL part of N2 and N3 with 162 Da corresponded to the molecular formula C_6_H_10_O_5_ (Table 3). This formula was consistent with a glucose loss and indicated that N2 and N3 were glycosylated forms of N1 and LS-A.

**Figure 8:**
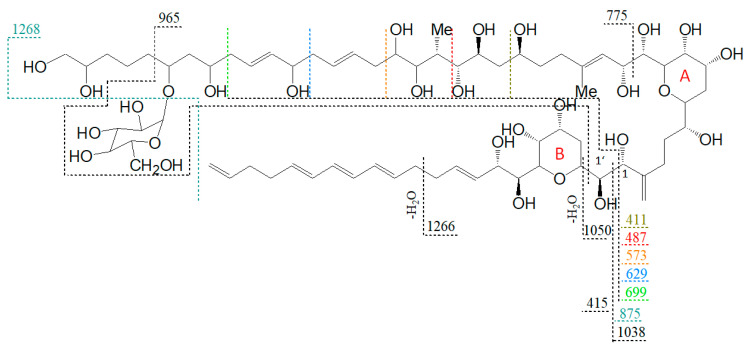
Proposed structure and fragmentation scheme of N3, the glycosylated form of N1.

A proposed structure and fragmentation scheme for N2, which includes the location of the hexose unit, is shown in Figure 8.

**Figure 9:**
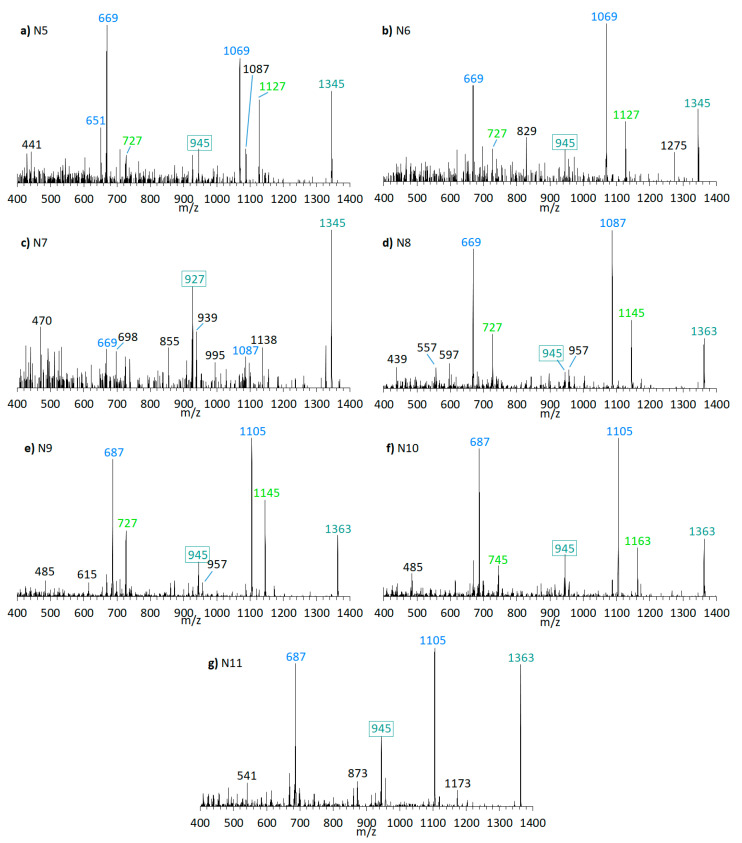
CID spectra of new compounds found in *A. carterae* strain CCMP1314. (**a**) N5, t_R_ = 2.17 min; (**b**) N6, t_R_ = 2.72 min; (**c**) N7, t_R_ = 3.02 min; (**d**) N8, t_R_ = 2.19 min; (**e**) N9, t_R_ = 2.78 min; (**f**) N10, t_R_ = 2.84 min; (**g**) N11; t_R_ =2.91 min. t_R_ = retention time.

**Figure 10:**
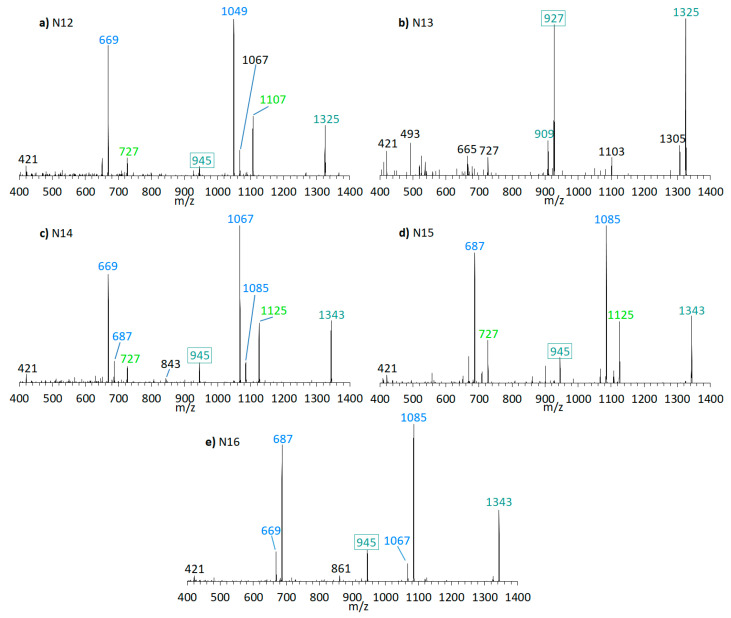
CID spectra of new compounds found in *A. carterae* strain DN241EHU. (**a**) N12, t_R_ = 2.21 min; (**b**) N13, t_R_ = 3.08 min; (**c**) N14, t_R_ = 2.23 min; (**d**) N15, t_R_ = 2.83 min; (**e**) N16, t_R_ = 2.97 min. t_R_ = retention time.

Furthermore, the presence of the already known AM-18 (*m*/*z* 1381 [M + Na]^+^) was confirmed in *A. carterae* strain CCMP1314 as well as AM-A (*m*/*z* 1361 [M + Na]^+^) in *A. carterae* strain DN241EHU. In this strains, twelve new compounds were detected in total (Figure 9 and Figure 10). CID spectra of the new compounds N7 (Figure 9c) and N13 (Figure 10b) correspond to the LP-D type, whereas the other new compounds showed a structural pattern of AM-18 with several large peaks resulting from the cleavages in the vicinity of the carbonyl group. Many of the newly detected AM candidates in strains CCMP1314 and DN241EHU showed minor mass differences with respect to AM-A and AM-18, respectively, such as mass reductions of 18 or 36 Da to AM-18 or AM-A. Moreover, the same fragment masses often appeared in different spectra, but their relative abundances varied greatly.

### 2.6. Selected Reaction Monitoring (SRM) Method

An LC-MS/MS method was developed, which included the 37 known AM as well as the new AM candidates found in this study (Table 4). This method was applied to different *Amphidinium* strains, which resulted in the detection of 27 different analogs and led to the AM profiles mentioned below.

### 2.7. Amphidinol Profiles

A high diversity of AM variants was found in strain ACRN02. The main components were LS-A and N1 with together accounted for almost 90% of total AM content, while N3 and N2 occurred in significantly lower abundances (N3: 4%, N2: 1%). In addition, LP-D and LP-B/C as well as N6, a substance similar to LP-B/LP-C, were detected. Two substances occurring in very small amounts (together < 1%) were putatively identified as AM-4 and CAR-E. With a total AM content of approximately 3680 fg cell^−1^, the strain ACRN02 was found to have the highest total toxin content within the samples tested (Table 5). Since all quantitation results referred to the LP-D standard, they were expressed as LP-D equivalents and have to be regarded estimates. In ACRN03 only LS-A and LP-D could be detected in traces (total content 3 fg cell^−1^). In the *A. carterae* strain CCMP1314, a total AM amount of 1213 fg cell^−1^ was determined. AM-A and AM-B accounted for 72% of the total AM content in this strain, while AM-22 accounted for only 4%. The rest was distributed among the novel compounds N5–N11, with N11 being the most abundant of this group, with 15%. The analysis of *A. carterae* strain DN241EHU provided a total AM content of 475 fg cell^−1^. AM-A and AM-B were determined in relative abundances of 25% and 6%, respectively. AM-22 contributed. 2% to the total AM content. The remaining portion of about 67% was attributed to the novel compounds N12–N16, with N16 accounting for a particularly high proportion of 30% of the total AM content of this strain. No AM were detected in the Brazilian *A. carterae* strain A01BR, in *A. massartii* strain AA39, in *A. operculatum* strain AA60, and in the *Amphidinium* sp. strain AA40 from Mexico.

The limit of detection defined as signal-to-noise ratio = 3 for LP-D in the SRM mode was determined as 2.9 pg on-column, which corresponds to 0.08 fg cell^−1^, based on a cell number of 10^7^ (roughly applicable to ARCN02, ACRN03, CCMP1314, DN241EHU, and AA39). The lower available cell number of strains AA40 and AA60 resulted in a cellular higher detection limit of 0.29 fg cell^−1^ (AA40) and 1.62 fg cell^−1^ (AA60), respectively. The available cell number of A01BR resulted in a detection limit of 0.0029 fg cell^−1^.

### 2.8. Hemolytic Activity

Hemolytic assays with erythrocytes resulted in the following EC_50_ values for each of the strains: 9.95 × 10^6^ ± 1.25 × 10^6^ pg of saponin per well (A01BR), 12.25 × 10^6^ ± 1.42 × 10^6^ pg per well (ACRN02), 15.15 × 10^6^ ± 0.74 × 10^6^ pg per well (ACRN03), 11.78 × 10^6^ ± 0.52 × 10^6^ pg per well (CCMP1314) and 8.98 × 10^6^ ± 0.92 × 10^6^ pg per well (DN241EHU). An equivalent saponin potency (pg per *A. carterae* cell) was calculated by dividing the EC_50_ for saponin by the EC_50_ for *A. carterae*. The corresponding equivalent saponin potency values for each of the strains were the following: 34 ± 3.5 pg cell^−1^ (A01BR), 80.6 ± 11.3 (ACRN02), 245.2 ± 8.4 (ACRN03), 208.8 ± 4.2 (CCMP1314) and 355.6 ± 16.1 (DN241EHU). These results suggested the presence of hemolytic AM in the methanolic extracts.

## 3. Discussion

### 3.1. Ionization and Fragmentation of Amphidinols

Several AM adducts have been described in the literature: The sodium adduct (+Na^+^) was used to identify AM-A [23], the hydrogen adduct (+H^+^) for AM-20 [14], and ammonium adduct (+NH_4_^+^) for AM-22 [16]. However, for a detection method that is both broadly applicable and allows for the comparison of ion intensities, it is highly advantageous to focus on one adduct form. This should preferably be the adduct form that occurs at the highest abundance and thus allows a high sensitivity. In the case of LP-D and the structurally closely-related karlotoxins [22], this applies to the sodium adduct (Figure 1). The stabilizing effect of the sodium ion [24] is the reason for the need for relatively high collision energies of up to 85 eV for the fragmentation of AM in the collision cell of the tandem mass spectrometer. Using the high-resolution CID spectrum of LP-D, it could be shown for the first time that almost all relevant fragments are associated with the C1′/C1 cleavage.

**Figure 11:**
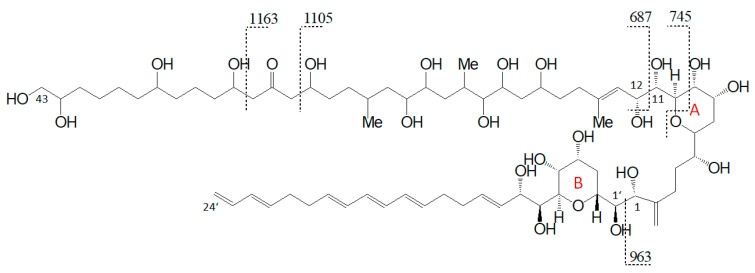
Fragmentation scheme of AM-18 according to Nuzzo et al. [7] with the newly proposed carbon numbering system.

With regard to the deviating fragmentation patterns of AM-18 and AM-A and their sulfated forms, AM-19 and AM-B described in Section 2.3, only one fragmentation scheme for AM-18 has been presented in the literature by Nuzzo et al. (2014) [7] (Figure 11). This scheme shows some differences to the one presented in this work (Figure 6), which makes an exact comparison necessary.

Nuzzo et al. (2014) [7] suggested cleavage sites in both β-positions of the carbonyl group at C31 flanked by a β-hydroxyl group on each side (C29 and C33) that lead to two of the three most abundant fragments (*m*/*z* 1163 and *m*/*z* 1105). These considerations are consistent with the scheme of this work (Figure 6). The third high abundant fragment at *m*/*z* 687 is explained by Nuzzo et al. (2014) with a C11/C12 cleavage, while the lower abundant fragment *m*/*z* 745 is explained with a cleavage of the tetrahydropyran ring A between C10 and the ether function (Figure 11). Furthermore, no occurrence of fragment pairs is reported in this scheme. However, a closer look at the HRMS data recorded by Nuzzo et al. (2014) [7] led to an alternative proposal for the fragments *m*/*z* 745 and *m*/*z* 687. Nuzzo et al. (2014) suggested a composition of C_37_H_70_Na^+^O_13_ and C_35_H_68_Na^+^O_11_, respectively (Table 6 (a)). According to the newly suspected fragmentation scheme, the fragments *m*/*z* 745 and *m*/*z* 687 are consistent with the elemental compositions of C_36_H_66_Na^+^O_14_ and C_33_H_60_Na^+^O_13_ (Table 6 (b)), which in turn correspond to the fragment pairs of *m*/*z* 1163 and *m*/*z* 1105 formed by the C1/C1′ cleavage and the loss of the lipophilic arm of AM-18 with a molecular weight of 418 Da (Figure 6).

The alternative proposal for the fragments *m*/*z* 745 and *m*/*z* 687 is further supported by the determined deviations between measured and calculated masses of about 35 ppm (Table 6 (b)), which fits the deviations between measured and calculated masses of the other described fragments (Table 6 (a)).

As AM-A, AM-19, and AM-B also possess a keto group with two β-hydroxyl groups in the hydrophilic arm as AM-18 [25], and these three AM differ only slightly from AM-18 in the lipophilic arm and/or sulfatation, AM-A, AM-19, and AM-B showed the same fragmentation pattern as AM-18 (Figure 5). Detailed spectra and fragmentation patterns of these substances are shown in Supplementary Materials Figures S7–S10.

Even though the C1/C1′ cleavage is common among all AM, the relative intensities of the respective fragments can vary from the base peak (i.e., 100% relative abundance) to hardly detectable. In practical terms, the relative intensities of characteristic fragments should be considered for the selection of suitable SRM transitions to ensure the highest sensitivity. This point is particularly important as the sensitivity of AM in tandem mass spectrometry is generally low in comparison to other phycotoxins and inappropriate transition selection would increase detection limits significantly. This specifically applies to AM with a carbonyl group flanked by two hydroxyl groups, which display low abundances of the C1/C1′ fragments (Figure 9 and Figure 10). Interestingly, no pair fragments were observed for LP-D in the negative ionization mode (Supplementary Materials Figure S11), which indicates completely different fragmentations as the positive.

### 3.2. Liquid Chromatography-Tandem Mass Spectrometry LC-MS/MS Methods

For many AM described in the literature, initially, no CID spectra were available as they have only been characterized by NMR (e.g., AM-3) [26]. However, structural information of all known AM from NMR and CID spectra recorded in this work revealed that the two tetrahydropyran rings and the beginning of the lipophilic and hydrophilic arm (C10′–C14) are conserved among AM (except for LP-A, missing a hydroxyl group at C8′ [9]), which leads to the conclusion that the C1′/C1 cleavage will occur in the fragmentation of these AM. This assumption, together with the structural information of AM, allows for the calculation of the *m*/*z* values of the fragments formed by the C1′/C1 cleavage, independent of the availability of mass spectrometric data of these compounds. However, it should be considered that sulfated AM always first eliminates the sulfate group before C–C cleavages occur. For example, AM-19 with *m*/*z* 1483 [M + Na]^+^ is a sulfated AM. A C1′/C1 cleavage would result in the fragment *m*/*z* 1065, however, this peak is hardly visible in the CID spectrum of AM-19 (Figure 5b). Instead, the cleavage between C1′/C1 occurs after the loss of the sulfate group, resulting in fragment *m*/*z* 945. The mass difference between the pseudo-molecular ion and the biggest fragment is 120 Da corresponding to NaHSO_4_. Accordingly, *m*/*z* values of the characteristic fragments were calculated for all AM included in this method and used for the development of a selected reaction monitoring (SRM) method for the targeted analysis and determination of all to date known AM (Table 4).

In addition to the development of the targeted SRM method, the C1′/C1 cleavage can also be used for the detection of unknown AM variants based on expected neutral losses (NL scan mode). The C1′/C1 cleavage creates a neutral fragment comprising the part from the C-1′ atom to the end of the lipophilic arm (for example AM-18: C1′ to C24′, Figure 11). Among the 37 known AM, there are only seven structural variants of the lipophilic arm. The molecular weights of these seven neutral fragments were calculated accordingly (Table 2) and included in the NL method. Even though this NL method is limited to the detection of novel AM containing one of the seven lipophilic arms, the probability of being able to detect an unknown AM is quite high because many AM variants include only a few structural changes and large parts of the molecules remain conserved. As in the case of the SRM method, sulfated AM must be considered separately in the NL method. Sulfated AM can only be detected successfully if 120 Da are added to the original neutral losses as the sulfate group is split off in addition to the lipophilic arm.

### 3.3. Method Advantages and Limitations

Quantification of phycotoxins depends on the availability of calibration standards. Even though isotope-labeled AM have been already used to study their synthetic pathway [27], no isotope-labeled AM are commercially available, which could ideally be used as internal standards for toxin quantification to avoid matrix effects. Alternatively, the standard addition method could be applied, but this method implies a high consumption of standard material, which is not available in most cases. Because of the impossibility of internal calibration and the unfeasibility of the standard addition method, AM were quantified by external calibration with an LP-D standard instead, and AM were expressed as LP-D equivalents, assuming similar molecular responses between LP-D and other AM. This assumption is justified by the fact that the fragments used for quantification are based on the same C1′/C1 cleavage site in all cases. However, it should be taken into consideration that, in the case of particularly large AM (e.g., AM-22, Figure 4a), AM with a carbonyl group (e.g., AM-A, Figure 5c), and/or sulfated AM (e.g., AM-B, Figure 5d), the resulting C1′/C1 fragment appears in significantly lower abundance than of LP-D. For this reason, all values given as LP-D equivalents are not exact determinations, but rather semi-quantitative estimates. In many cases, where analytical standards are not available, estimates may be sufficient for many scientific purposes, e.g., for the determination of toxin absence/presence, distribution in field samples, or relative variation in profile and quantitative composition in laboratory tests.

In the present SRM method, only a single transition was defined for each AM. In addition, some AM have identical masses and possess remarkably similar structures (e.g., AM-3 and AM-9). Therefore, in some cases, it is essential to record a CID spectrum for the unambiguous identification of the respective AM. In rare cases, however, even a CID spectrum of the sodium adduct is not sufficient to distinguish between two substances. For example, LP-B and LP-C differ only in the position of two hydroxyl groups on the lipophilic arm, which does not fragment under the conditions mentioned above. A CID spectrum in negative mode could make possible an exact distinction, like it was the case, for example, in the characterization of AM-7 [13]. Almost all C–C bonds broke so that a complete determination of the lipophilic arm was also possible. Moreover, the SRM method easily can be expanded by an unlimited number of AM. For this purpose, only the structure of the AM has to be known to calculate the *m*/*z* value of the fragment resulting from the C1′/C1 cleavage.

### 3.4. Characteristics of New Amphidinols

In summary, the known and newly discovered AM can be assigned to one of two groups. The first group describes LP-D-like substances whose CID spectra are usually characterized by a single prominent peak. The second group contains mainly AM with a carbonyl group, which has a considerable influence on fragmentation and results in several prominent peaks. Sulfated AM appear in both groups, and sulfatation is recognized by a pseudo molecular ion with low relative intensity and a 120 Da lighter fragment. The spectra of substances N1, N2, N3, N4, N7 and N13 (Figure 7, Figure 8, Figure 9 and Figure 10). can be assigned the LP-D group without sulfur, as the fragments formed by the C1′/C1 cleavage have the highest abundance and no peak with a difference of 120 Da to the molecule ion appears.

N1 (*m*/*z* 1267 [M + Na]^+^) shows a very similar spectrum to LS-A (*m*/*z* 1295 [M + Na]^+^, Figure 4b) and differs from LS-A by the absence of two methyl(ene) groups or one ethyl(ene) group. Both spectra are characterized by several identical pair fragments (*m*/*z* 1091 and 699, *m*/*z* 1021 and 629, *m*/*z* 965 and 573, *m*/*z* 803 and *m*/*z* 411), which have a difference of *m*/*z* 392. The common fragment *m*/*z* 415 of LS-A and N1, which represents the sodium adduct of C1′–C22′, also indicates that the missing alkyl groups must be located on the hydrophilic arm.

The lower fragments mentioned above (*m*/*z* 699, *m*/*z* 629, *m*/*z* 573, *m*/*z* 411) are also present in the spectra of N2 and N3 (Figure 7b,c), though the corresponding pair fragments are missing (except for *m*/*z* 411 in N3). In addition, the masses of the pseudo-molecular ions of N1 and LS-A are present in low abundance in the spectra of N2 and N3, respectively, and their mass differences are 162 Da, equivalent to C_6_H_10_O_5_ as determined by HRMS (Table 3). This is a strong indication that N2 and N3 are glycosylated forms of N1 and LS-A, respectively. The high abundance of the C1′/C1 fragments *m*/*z* 1038 and *m*/*z* 1066 that include the hexose unit indicates that this unit is located on the hydrophilic arm of both molecules. The glycosylation bond seems to be more stable than the C1′–C1 bond so that the C1/C1′ cleavage is preferred over the elimination of the hexose.

N4 (*m*/*z* 1315 [M + Na]^+^, Figure 7d) shows a difference of *m*/*z* 440 between the pseudo-molecular ion and the C1′/C1 fragment (*m*/*z* 875), which, together with the fragment *m*/*z* 463, indicates a neutral loss type 5 (Table 2), which otherwise only occurs in LP-B and LP-C. In addition, LP-B/C and N4 share the pair fragment *m*/*z* 1069 and *m*/*z* 629. Interestingly, LP-B/C and N4 among the analyzed strains were only found in strain ACRN02, which supports the hypothesis that AM variability within a strain is based mostly on variations of one core structural element and accordingly structural variability within strains is lower than across different strains. However, a crucial difference between N4 and LP-B/C is the occurrence of many fragments between *m*/*z* 450 and 570 in N4. Such an appearance of many fragments in this relatively narrow mass range could not be detected so far in other measurements of sodium adducts of AM, which indicates a hitherto unknown modification of the hydrophilic arm.

N7 (*m*/*z* 1345 [M + Na]^+^, Figure 9c) was detected in CCMP1314, while N13 (*m*/*z* 1325 [M + Na]^+^, Figure 10b) was detected in DN241EHU. Both substances are the only ones in the respective strains that fragment according to the LP-D group with a single large peak besides the pseudo-molecular ion. This suggests that these AM candidates, compared to others found in these strains, do not possess a carbonyl group flanked by hydroxyl groups. Together with N5, N6, N8, N9, N10, N11, N12, N14, N15, and N16 (Figure 9 and Figure 10), N7 and N13 differ in 18 or 36 Da from AM-18 (*m*/*z* 1381 [M + Na]^+^) and AM-A (*m*/*z* 1361 [M + Na]^+^), which also were present in high abundances in the respective strains. Mass differences of 18 and/or 36 Da often can be attributed to in-source water losses of parent compounds, but this can be excluded for the new AM reported here as these AM have different retention times and thus must be independent, but closely related variants of AM-18 and AM-A, respectively.

In terms of biosynthetic pathways, the comparison of the AM profiles of strain CCMP1314 and DN241EHU is interesting. The main compound of strain CCMP1314 is AM-18 and of strain DN241EHU is AM-A. Both AM share the hydrophilic arm and the core unit but differ in the lipophilic arm, which in AM18 is longer and has a higher number of double bonds than AM-A. The minor AM of both strains (N5, N8, N9, N11 in of CCMP1314 and N12, N14, N15, N16 in DN241EHU) showed the same modifications of their respective main AM. N5 (CCMP1314, Figure 9b) and N12 (DN241EHU, Figure 10a have similar retention times (2.17 min and 2.21 min) and an almost identical peak pattern with similar intensities. N12 differs from N5 mainly by the fragments *m*/*z* 1049, *m*/*z* 1067, *m*/*z* 1107, and *m*/*z* 1325, which are each *m*/*z* 20 lighter than the respective fragments from N5. The same applies to N8 (2.19 min) and N14 (2.23 min), where the lighter fragments *m*/*z* 669, *m*/*z* 727, and *m*/*z* 945 occur in both substances, while the heavier fragments of N8 (*m*/*z* 1087, *m*/*z* 1145, *m*/*z* 1363) are increased by *m*/*z* 20 regarding N14 (*m*/*z* 1067, *m*/*z* 1125, *m*/*z* 1343). In addition, N14 shows the fragment *m*/*z* 1085, for which there is no visible corresponding fragment in N8. N9 (2.78 min) and N15 (2.83 min) also share a similar peak pattern, including fragments *m*/*z* 687, *m*/*z* 727, and *m*/*z* 945 in nearly the same intensities. The heavier fragments showed again a difference of 20 Da. Last, N11 (2.91 min) and N16 (2.97 min) showed a similar spectrum with only one high intense fragment (*m*/*z* 1105 for N11, *m*/*z* 1085 for N16) besides the pseudo-molecular ion. As a result, a corresponding fragment with *m*/*z* 727 or *m*/*z* 745, which occurred in the other substances, is also missing. For the compounds N6 and N10, detected in the strain CCMP1314, which were detected only in very small amounts, no corresponding substances could be detected in DN241EHU, which could possibly be due to the overall lower AM content of DN241EHU. In summary, the similarity between the AM of strains CCMP1314 and DN241EHU indicates a conserved biosynthetic pathway in both strains that only seem to differ in their polyketide precursor.

Due to the relatively low number of fragments formed by sodium adducts of AM under CID conditions, only limited structural information could be obtained from the spectra. On the other hand, the low number of fragments and the characteristic cleavage sites lead to typical fragment patterns of AM, which facilitated the recognition and the attribution of unknown compounds to the AM compound family and the AM-like CID spectra of N1 to N16 served as strong evidence that the newly discovered substances indeed are AM. Nonetheless, the precise structures of the new substances can only be verified by nuclear magnetic resonance spectroscopy (NMR). For NMR, purified substances are required in sufficiently high amounts, which were not available for this study.

### 3.5. Amphidinol Profiles

AM were detected in four of the eight examined strains. The AM profiles of these strains (ACRN02, ACRN03, CCMP1314, and DN241EHU) were dominated by one or two AM as the main components and the other AM variants only occurred in small amounts. A similar pattern of karlotoxins that are structurally very similar to AM was found in different *Karlodinium* strains [22]. However, the quantitative expression of AM in *Amphidinium* resulting in varying AM cell quotas seems to be variable as ACRN02 in this study showed a higher number and higher AM cell quota than ACRN03 (Table 5), which contrast with a previous study that showed that the latter produce large amounts of LP-D and LS-A [11].

Martínez et al. (2019) previously analyzed the *A. carterae* strain CCMP1314 and detected AM-18, AM-19, AM-22, and other, not further characterized AM-like compounds [16]. This study confirmed the presence of AM-18, AM-19, and AM-22 and additionally detected compounds N5–N11, likely the AM-like compounds reported earlier [16]. Similarly, AM-A and AM-B were reported for the *A. carterae* strain DN241EHU [28], but no further components were detected. The NL method applied here revealed the presence of N12–N16, in addition to AM-22 and the previously reported AM-A and AM-B in strain DN241EHU (Table 3). Interestingly, compounds N5–N11 of strain CCMP1314 followed the same fragment pattern of the major compound AM-18 of this strain (Figure 9). The same was observed for strain DN241EHU with AM-A as the main compound and N12–N16 as new AM, which presented a similar fragmentation pattern as AM-A (Figure 10). This indicates that the chemical variability within one strain is constrained to minor changes of a conserved core structure.

No AM were detected in strains AA39, AA40, AA60, and A01BR. Only A01BR was identified as *A. carterae,* while AA39 was identified as *A. massartii*, AA60 as *A. operculatum* and AA40 as *Amphidinium* sp. To the best of our knowledge, no AM production in *A. massartii* and *A. operculatum* has been reported so far, which leads to the assumption that not all species of the genus *Amphidinium* produce AM. Nevertheless, no AM might have been found in these strains due to the fact that the neutral loss method does not detect unknown AM derivatives with modifications of the lipophilic arm. On the other hand, these strains did not show peaks in the mass range most common to AM (*m*/*z* 1100 and 1700) in high-resolution full-scan mode, indicating that in fact, no AM-like substances were present. Another explanation for the absence of AM in the Mexican strains AA39, AA40, AA60 may be the available biomass ranging from 0.54 × 10^6^ cells (AA60) over 3 × 10^6^ cells (AA40) up to 9 × 10^6^ cells (AA39), which was lower as for the strains, in which AM could be detected (approximately 10 × 10^6^ cells).

## 4. Materials and Methods

### 4.1. Amphidinium Strains

General information on *Amphidinium* strains used in this study are shown in Table 7. The Mexican strains AA39 and AA60 were identified as *A. massartii* and *A. operculatum*, respectively (in process of publication). AA40 is reported as *Amphidinium* sp. while the detailed morphological and molecular designation is performed.

### 4.2. Culture Conditions and Culture System

The three Mexican strains were maintained in 70 mL plastic culture flasks in a temperature-controlled culture room at 24 °C, a photon flux density of 70 µE m^−2^ s^−1^ on a 16:8 h light:dark photocycle in a natural seawater medium prepared with sterile-filtered (0.2 µm VacuCap filters, Pall Life Sciences, Dreieich, Germany) Antarctic seawater (salinity: 34, pH adjusted to 8.0) and enriched with 1/10 strength K-medium [29]. For toxin analysis, the strains were grown in 300 mL plastic flask under the standard culture conditions outlined above. At the stationary phase, cell density (ranging from 3 to 30 × 10^3^ cells mL^−1^) was determined by settling Lugol’s fixed samples and counting > 400 cells under an inverted microscope.

Inocula of strains A01BR, ACRN02, ACRN03, DN241EHU, and CCMP1314 were grown in flasks at 19 ± 1 °C placed in a thermostated chamber under a 12:12 h light:dark cycle. 58 W fluorescent lamps were used for illumination and the irradiance at the surface of the culture flasks was 60 µE m^−2^ s^−1^. The following culture medium formulations were used: (i) f/2 for the strains CCMP1314 and DN241EHU [30]; (ii) modified K medium for the strains A01BR, ACRN02, and ACRN03 [11]. Media prepared from natural, filter-sterilized (0.22 μm) Mediterranean seawater was used to maintain the inocula. Inocula were acclimated to the illumination level and culture media above mentioned for more than a year. Monocultures of A01BR, ACRN02, ACRN03, DN241EHU, and CCMP1314 were conducted at 19 ± 1 °C in methacrylate bubble column photobioreactors (BCs) similar to those earlier reported [31]. Briefly, BCs with a volume of 3.0 L (2 m high, 0.044 m internal diameter) were positioned vertically at a distance of 0.3 m from the light source. Illumination was provided by 58W fluorescent lamps emitting an average irradiance at the surface of the columns of 60 µE m^−2^ s^−1^. Cultures were pneumatically agitated by filter-sterilized air sparged through a nozzle with 1.5 mm internal diameter. Prior to use, the BCs were sterilized by filling the vessels and associated pipework with filtered seawater, adding commercial bleach (~3 mL per L of water), mixing gently, and letting stand (no mixing or aeration) for several hours. Once the treatment had been completed, the bleach was neutralized by adding a solution of sodium thiosulfate (250 g sodium thiosulfate (Na_2_S_2_O_3_·5H_2_O) dissolved in 1 L of water; 1 mL of this solution was added for each 4 mL of the bleach used). The culture medium was prepared as described above. The fresh medium was inoculated with 10% *v*/*v* of an inoculum containing algal cells in the late exponential growth phase. Cultures were operated in batch mode.

A Cell Lab Quanta SC flow cytometer (Beckman Coulter Inc., Brea, CA, USA) was used to quantify the cell density in the cultures. At least 60,000 cells were counted per sample. Triplicate samples were measured, and the data were averaged. The flow rate was kept at a moderate setting (data rate = 600 events s^−1^) to prevent interference between cells.

### 4.3. Hemolytic Activity

The assay for the detection of hemolytic activity of the methanolic extracts of strains A01BR, ACRN02, ACRN03, DN241EHU, and CCMP1314 was performed as described by Eschbach et al. (2001) [32]. In short, a sample of the methanolic extract was placed in a microwell and air-dried. Erythrocytes from defibrinated sheep blood were used at a concentration of 45 × 10^6^ cells per well. Erythrocytes incubated in Mediterranean seawater served as negative controls, and the positive control, or 100% hemolysis, was obtained using distilled water. The dose-response curves (percentage of hemolysis (PH) vs. log of number of *A. carterae* cells per well (x)) were fitted to the Hill equation by nonlinear regression:

(1) PH= PHmin+ PHmax − PHmin1+(xEC50)η 

where PH_max_ is the maximum percentage of hemolysis equal to 100%, EC_50_ is the number of *A. carterae* cells per well giving 50% hemolysis and η is the Hill slope. The Hill curve was also applied to hemolysis experiments with Saponin (Sigma Aldrich, 47036, CAS nº 8047-15-2, Saint Louis, MO, USA). 12 data points were used for both, sample and saponin curve. The range of concentrations was 0–229 µg saponin/well and 0–10^5^ cells/well.

### 4.4. Chemical Analysis

#### 4.4.1. Harvest of *Amphidinium* Cells

Cells of the Mexican strains were harvested by centrifugation at 5250× *g* and 10 °C for 15 min in an Allegra X-15 R laboratory centrifuge (Beckman Coulter GmbH, Krefeld, Germany) and the supernatant was removed. After a short resuspension, the samples were pelleted again at 16,100× *g* and 10 °C for 15 min and the supernatant was decanted.

Once the stationary phase of strains A01BR, ACRN02, ACRN03, DN241EHU, and CCMP1314 was reached, the cultures were deposited in 50 mL Falcon tubes and centrifuged at 2500× *g* for 5 min at room temperature in a benchtop centrifuge (Beckman Coulter, model Allegra 25R, Madrid, Spain) using a rotor (swing-out head) with a maximum radius of 13.7 cm (max RCF = 15,300× *g*). These centrifugation conditions were within the operating window previously determined for *Amphidinium carterae* where maximum cell recovery and cell separation-cell integrity are guaranteed [33]. The pellets were gently washed with distilled water. Cells were again pelleted, and the supernatants were carefully removed. The frozen biomass pellets were dried in a vacuum freeze-dryer (Cryodos 50, Telstar, Madrid Spain) for 48 h.

#### 4.4.2. Extraction of Amphidinols

##### AA39, AA40, AA60

The cells of *Amphidinium* sp. strains AA39, AA40, and AA60 were extracted by addition of 500 µL methanol and 0.9 g Lysing Matrix D and suspension on a vortexer followed by lysis by reciprocal shaking at maximum speed (6.5) for 45 s in a Bio 101 FastPrep device (Thermo Savant, Illkirch, France). Solid cell residues and Lysing Matrix D were then separated from the methanol extract by centrifugation at 16,100× g and 10 °C for 5 min. The methanol extract was transferred to a centrifugation filter (Ultrafree, Millopiore, Eschborn, Germany) with a pore size of 0.45 µm and centrifuged at 16,100× *g* and 10 °C for 1 min. Finally, the filtrate was transferred to an HPLC vial and sealed with a lid containing a silicone septum. Samples were stored at −20 °C until analysis.

##### ACRN02, ACRN03, CCMP1314 and DN241EHU

The dried biomasses were extracted three times with 15 mL methanol (methanol:biomass ratio of 30 (*v*/*d.w*.)). The extraction process was ultrasonication-aided by an ultrasonic probe-type device (UP200S, Hielscher Ultrasonics™; 200 W, 24 kHz, 4 min, Hielscher, Teltow, Germany). The sonication power was 50% of full power, and the pulse control was set at 0.5. The sonicated suspensions were centrifuged (4000× *g*, 10 min, 10 °C), the methanolic supernatants were recovered and filtered (0.22 μm pore size membrane filter) to remove debris. For every strain, aliquots equivalent to 10^7^ cells were dried under an N_2_ stream and used as a raw material in the LC-MS/MS-based detection method. After shipment from University of Almería (Spain) to Alfred-Wegener-Institute (Germany), the dry extracts were dissolved in 400 µL methanol, each tube was washed twice with additional 400 µL and combined extracts of each strain were transferred to an HPLC sample vessel. Methanol was completely evaporated under a fume hood with nitrogen. The residues were then dissolved with 100 µL methanol and transferred to a centrifugation filter with a pore size of 0.45 µm. The HPLC sample tubes were rinsed again with 50 µL methanol and the resulting extracts were also transferred to the centrifugation filter. Extracts were centrifuged at 16,100× *g* and 10 °C for 1 min to remove suspended solids. Finally, the filtrates were transferred to conical HPLC sample tubes and closed with a lid containing silicone septum. Samples were stored at −20 °C until measurement.

##### A01BR

After adding 5 mL methanol to the pellet of the *A. carterae* strain A01BR, it was digested for 1 min with an ultrasonic rod (SONOPLUS HD2070 MS72, Bandelin electronic, Berlin, Germany) at 70% cycle and 72% power. By short vortexing, the cells located on the wall of the tube were rewound back into the solution and the ultrasonic treatment was repeated under the same settings. The sample was then centrifuged at 5250× *g* for 5 min (Allegra X-15 R laboratory centrifuge, Beckman Coulter GmbH, Krefeld, Germany). The supernatant was divided in equal parts into four HPLC vial and sealed with silicone septum lids. These were stored at −20 °C until further use. From one of the aliquots, 300 µL were taken and transferred to a 0.45 µm centrifugation filter. After centrifugation filtration at 16,100× *g* and 10 °C for 1 min, the sample was placed in a conical HPLC vial and closed with a lid with silicone septum. Since the measurement of the centrifugation-filtered sample did not yield positive results, the four extracts were combined and concentrated. For this purpose, the extracts were combined in a 15 mL centrifuge tube (approx. 4.5 mL) and the methanol was evaporated stepwise with nitrogen. The residues were finally dissolved with 500 µL methanol and divided equally between two HPLC sample tubes. The centrifuge tube was then rinsed twice with 100 µL methanol each and this was also divided between the two HPLC sample tubes. From one of the two aliquots, 200 µL were then centrifugation-filtered according to the previously mentioned scheme and stored at −20 °C until measurement.

#### 4.4.3. LC-MS/MS Analysis

Ultra-Performance Liquid Chromatography (UPLC^®^) coupled with tandem quadrupole mass spectrometry was used for AM analysis. The UPLC system comprised an AQUITY UPLC column oven, an AQUITY I UPLC class autosampler (operating temperature 8 °C) and an AQUITY I UPLC class binary pump. Separation of the methanol extract was performed after injection of 0.5 µL sample by a Purospher^®^STAR RP-18 endcapped (2 µm) Hibar^®^ HR 50-2.1 UPLC column (Merck, Darmstadt, Germany), which was maintained at 40 °C. A 0.5 µm OPTSSOLV ^®^ EXP™ precolumn (Sigma-Aldrich, Hamburg, Germany) was used. This system was coupled to a Xevo^®^ TQ-XS mass spectrometer (Waters GmbH, Eschborn, Germany). Data were collected and analyzed with Masslynx software (version 4.2, Waters). The flow rate was 0.6 mL min^−1^, and gradient elution was performed with two eluents, where eluent A was water and eluent B was acetonitrile/water (9:1 *v*/*v*), both containing 6.7 mM ammonia. The gradient was as follows: The measurement was initialized with 70% A until 1.5 min, followed by a linear gradient to 10% A until 3.5 min. This condition was maintained until 4.0 min and the gradient was then returned to initial conditions until 4.1 min. These settings were retained up to 5.0 min (= total running time). For NL-, CID- and SRM-scans, the collision energy for sulfated AM and AM with carbonyl group was 75 eV, while for AM with a similar structure to the standard substance LP-D, a collision energy of 85 eV was used. All measurements were performed in positive mode. The used neutral losses and transitions for SRM are given in Table 2 and Table 4. CID spectra were recorded of the [M + Na]^+^ adduct ions of all available compounds. Further mass spectrometric parameters are given in Table 8.

#### 4.4.4. High-Resolution Mass Spectrometry

LC-HRMS analysis was performed with a Vanquish UPLC system coupled to a Q-Exactive Plus mass spectrometer, using a heated electrospray ionization source (both Thermo Fisher Scientific, Bremen, Germany). Separation was performed using a binary solvent gradient with solvent A = 0.1% formic acid in ultrapure water and solvent B = 0.1% formic acid in methanol with a flow rate of 0.4 mL min^−1^ on a C_18_ column (C18 BEH, 100 × 2 mm, 1.7 µm particle size, ACQUITY Waters, Eschborn, Germany, equipped with guard-column) at 32 °C. The gradient program was as follows: T_0 min_: B = 5%, T_0.1 min_ B = 5%, T_3 min_: B = 99%, T_5min_: B = 99%; T_5.2 min_: B = 5%. The column was equilibrated for 1 min between samples. MS^2^ spectra were acquired by the data-dependent (dd-MS²) or data-independent (DIA) mode. dd-MS² experiments were performed with a full scan at resolution (RES) = 140,000 followed by dd-MS² at NCE (normalized collision energy) of 45, automatic gain control (AGC) of 2e5, isolation window (IW) of 1.0 *m*/*z*, injection time (IT) of 50 ms, a RES = 70,000 full width at half maximum (FWHM) an inclusion list of accurate masses. DIA experiments also utilized an inclusion list of accurate masses (four entries maximum) at a resolution of 280,000 (*m*/*z* 200), stepwise normalized collision energy (NCE) 40, 50 and 55, automatic gain control (AGC) target of 2e5 and isolation window of 0.8 *m*/*z*. The spray voltage for all experiments was 3.5 kV. The capillary temperature was set to 256 °C, the aux gas to 413 °C (11) and the sheath gas to 48. Positive Ion Calibration Solution (Pierce, Thermo Fisher Scientific) was used for the calibration of the instrument. Molecular Assignment for the molecular formulas of the [M + Na]^+^ ions were accepted when within the mass tolerance range of the instrument’s error (<1 ppm).

## 5. Conclusions

The thorough analysis of the fragmentation patterns of AM and related compounds allows for the identification of new structural variants of this toxin group by mass spectrometry. This is particularly useful as NMR, the classical method for structural elucidation of unknowns requires relatively high amounts of purified compounds, which in many cases are difficult to obtain. In the case of AM, the number of unidentified structural variants and therefore the number of compounds with varying degrees of biological activity may be high and depending on the producing species and geographic origin. Further, the molecular size of these polyketides allows for a high structural variability as seen in other classes such as yessotoxins [34] and prymnesins [35]. The high structural similarity of AM to karlotoxins suggests that AM may be involved in predation and grazing defense by membrane lysis of other protistan organisms. The biological activity of the glycosylated AM first described here is especially intriguing for further investigations. Whereas other more polar AM such as the sulfated forms were not biologically active in in vitro, nothing is known on the toxicity of glycosylated AM. One hypothesis for the biological inactivity of sulfated and most likely also of glycosylated AM may be that these polar AM are biosynthetic precursors that prevent self-intoxication of the producing cells.

## Figures and Tables

**Table 1:** Exact masses, elemental composition, and error between empirical and theoretical masses of LP-D fragments.

*m*/*z* Observed	Elemental Composition	Calculated Exact Mass [Da]	±Da	±ppm
1329.7548	C_66_H_114_NaO_25_	1329.7549	0.0001	0.08
1311.7440	C_66_H_112_NaO_24_	1311.7441	0.0001	0.09
1125.6196	C_56_H_94_NaO_21_	1125.6207	0.0011	0.95
1055.5768	C_52_H_88_NaO_20_	1055.5769	0.0001	0.13
1037.5656	C_52_H_86_NaO_19_	1037.5661	0.0005	0.48
999.5144	C_48_H_80_NaO_20_	999.5147	0.0003	0.34
911.4613	C_44_H_72_NaO_18_	911.4616	0.0003	0.33
903.5291	C_44_H_80_NaO_17_	903.5293	0.0002	0.22
885.5189	C_44_H_78_NaO_16_	885.5190	0.0001	0.16
837.4247	C_41_H_66_NaO_16_	837.4249	0.0002	0.19
699.3930	C_34_H_60_NaO_13_	699.3932	0.0002	0.23
681.3824	C_34_H_58_NaO_12_	681.3826	0.0002	0.29
629.3509	C_30_H_54_NaO_12_	629.3513	0.0004	0.63
611.3403	C_30_H_52_NaO_11_	611.3407	0.0004	0.71
573.2885	C_26_H_46_NaO_12_	573.2887	0.0002	0.34
485.2361	C_22_H_38_NaO_10_	485.2363	0.0002	0.34
449.2148	C_22_H_34_NaO_8_	449.2151	0.0003	0.75
411.1987	C_19_H_32_NaO_8_	411.1995	0.0008	1.91

**Table 2:** Masses of different neutral loss (NL) types, which occur in previously discovered AM. In addition, the frequencies of the individual NL types in known AM and the masses of the sulfated form are shown.

#	Neutral Loss [Da]	Observed Frequency	Neutral Loss of Sulfated Forms [Da]
1	392.13	14/37	512.12
2	426.23	7/37	546.23
3	418.24	7/37	538.24
4	398.28	5/37	518.28
5	440.25	2/37	560.25
6	442.23	1/37	562.23
7	338.18	1/37	458.18

**Table 3:** Exact masses, elemental composition, and error between theoretical and empirical masses for LS-A and N1 to N3.

Toxin	*m*/*z* Observed	Elemental Composition	Calculated Exact Mass [Da]	±Da	±ppm
N1	1267.7164	C_64_H_108_Na^+^O_23_	1267.7174	0.0010	0.789
N2	1429.7694	C_70_H_118_Na^+^O_28_	1429.7702	0.0008	0.560
LS-A	1295.7481	C_66_H_112_Na^+^O_23_	1295.7487	0.0006	0.463
N3	1457.8007	C_72_H_122_Na^+^O_28_	1457.8015	0.0008	0.549

**Table 4:** Calculated transitions of already known and newly discovered AM. The fragmentation type according to the structure and known retention times are also given. (* = hypothesized transition; (M) = detected by Molina-Miras et al. [11]; (S) = detected by Satake et al. [14]; LP-D-type = A; carbonyl type = B; other type = C; no further groups = 0; sulfated = 1; glycosylated = 2).

Toxin	Q1 Mass (*m*/*z*)	Q3 Mass (*m*/*z*)	Retention Time (min)	Type	Toxin	Q1 Mass (*m*/*z*)	Q3 Mass (*m*/*z*)	Retention Time (min)	Type
AM-1 *	1511.8	973.6	-	A1	KAR-B *	1461.9	1063.6	-	B0
AM-2 *	1397.8	1005.6	-	C0	LP-A *	1277.5	753.5	-	A1
AM-3 *	1349.8	931.6	-	A0	LP-B	1343.8	903.5	2.23	A0
AM-4	1323.8	931.6	2.62	A0	LP-C	1343.8	903.5	2.23	A0
AM-5 *	1393.8	975.6	-	A0	LP-D	1329.7	903.5	2.00	A0
AM-6 *	1367.8	975.6	-	A0	LS *	1373.8	975.6	-	B0
AM-7 *	1253.6	741.5	-	A1	LS-A	1295.8	903.5	2.58	A0
AM-9 *	1349.8	931.6	-	A0	LS-B *	1265.6	753.5	-	A1
AM-10 *	1295.8	903.5	-	A0	SP *	1265.6	753.5	-	A1
AM-11 *	1499.8	987.6	-	C1	N1	1267.7	875.5	2.56	A0
AM-12 *	1425.7	913.6	-	A1	N2	1429.8	1037.6	2.52	A2
AM-13 *	1451.7	913.6	-	A1	N3	1457.8	1065.6	2.53	A2
AM-14 *	1287.6	741.5	-	A1	N4	1315.8	875.5	2.16	C0
AM-15 *	1185.7	759.5	-	A0	N5	1345.8	945.6	2.17	B0
AM-17 *	1305.7	815.5	-	A1	N6	1345.8	945.6	2.72	B0
AM-18	1381.8	963.6	2.71	B0	N7	1345.8	927.5	3.02	A0
AM-19	1483.8	945.6	2.20	B1	N8	1363.8	945.6	2.19	B0
AM-20 (M) *	1345.8	903.5	-	A0	N9	1363.8	945.6	2.78	B0
AM-20 (S) *	1652.5	1259.8	-	A0	N10	1363.8	945.6	2.84	B0
AM-21	1798.1	1405.9	-	A0	N11	1363.8	945.6	2.91	B0
AM-22 *	1667.9	1329.8	2.60	A0	N12	1325.9	945.6	2.21	B0
AM-A	1361.9	963.6	2.76	B0	N13	1325.9	927.5	3.08	A0
AM-B	1463.8	945.6	2.27	B1	N14	1343.9	945.6	2.23	B0
AMD-G *	1299.6	767.5	-	A1	N15	1343.9	945.6	2.83	B0
CAR-E	1421.9	1029.6	2.52	C0	N16	1343.9	945.6	2.97	B0
KAR-A *	1479.9	1081.6	-	B0					

**Table 5:** AM cell quotas [fg cell^−1^] expressed as LP-D equivalents of eight *Amphidinium* strains investigated in this study (- = not detected; * = abundance was too low for unambiguous identification).

Toxin	ACRN02	ACRN03	CCMP1314	DN241EHU	A01BR	AA39	AA40	AA60
AM-A	-	-	-	121	-	-	-	-
AM-B	-	-	-	28	-	-	-	-
AM-4 (*)	4	-	-	-	-	-	-	-
AM-18	-	-	601	-	-	-	-	-
AM-19	-	-	266	-	-	-	-	-
AM-22	-	-	49	11	-	-	-	-
CAR-E (*)	2	-	-	-	-	-	-	-
LS-A	1876	3	-	-	-	-	-	-
LP-B/C	53	-	-	-	-	-	-	-
LP-D	131	<1	-	-	-	-	-	-
N1	1412	-	-	-	-	-	-	-
N2	39	-	-	-	-	-	-	-
N3	149	-	-	-	-	-	-	-
N4	14	-	-	-	-	-	-	-
N5	-	-	12	-	-	-	-	-
N6	-	-	3	-	-	-	-	-
N7	-	-	14	-	-	-	-	-
N8	-	-	29	-	-	-	-	-
N9	-	-	53	-	-	-	-	-
N10	-	-	9	-	-	-	-	-
N11	-	-	177	-	-	-	-	-
N12	-	-	-	16	-	-	-	-
N13	-	-	-	78	-	-	-	-
N14	-	-	-	29	-	-	-	-
N15	-	-	-	43	-	-	-	-
N16	-	-	-	149	-	-	-	-
∑	3680	3	1213	475	0	0	0	0

**Table 6:** Calculated and observed masses of selected peaks of the HRMS spectrum of AM-18. The contents in italics originate from Nuzzo et al. [7] (**a**) fragments according to the fragmentation scheme by Nuzzo et al.; (**b**) fragments according to the newly suspected fragmentation scheme.

	*m*/*z* Observed	Elemental Composition	Calculated Exact Mass [Da]	±Da	±ppm
(**a**)	*1381.7755*	*C_71_H_122_Na^+^O_24_*	1381.8218	0.0463	33.506
	*1163.6303*	*C_60_H_100_Na^+^O_20_*	1163.6700	0.0397	34.116
	*1105.5908*	*C_57_H_94_Na^+^O_19_*	1105.6282	0.0374	33.827
	*963.5534*	*C_47_H_88_Na^+^O_18_*	963.5863	0.0329	34.143
	*745.4083*	*C_37_H_70_Na^+^O_13_*	745.4709	0.0626	83.973
	*687.3693*	*C_35_H_68_Na^+^O_11_*	687.4654	0.0961	139.789
(**b**)	*745.4083*	C_36_H_66_Na^+^O_14_	745.4345	0.0262	35.147
	*687.3693*	C_33_H_60_Na^+^O_13_	687.3693	0.0233	33.896

**Table 7:** Specifications of different *Amphidinium* strains analyzed in this study (m = morphological identification; g = genetic identification; n.s. = not specified).

	*Amphidinium*	Provider	Origin	Harvested Cells
ACRN02	*carterae* (n.s.)	Harmful Microalgae Culture Collection at IEO (Vigo, Spain)	La Réunion, Ind. Ocean	10 × 10^6^
ACRN03				10 × 10^6^
A01BR			Brazil	2.5 × 10^9^
CCMP1314	*carterae* (m + g)	Bigelow Laboratory (Maine, USA)	Falmouth Great Pond, USA	10 × 10^6^
DN241EHU	*carterae* (m + g)	Culture Collection of the Basque Country (Leioa, Spain)	Punta des Gas, Spain	10.3 × 10^6^
AA39	*massartii* (m + g)	Benthic dinoflagellate collection at UNAM, and CICESE (Mexico City, Mexico)	Baja California Sur, Mexico	9 × 10^6^
AA40	sp.			3 × 10^6^
AA60	*operculatum* (m + g)		Veracruz Reef System, Mexico	0.54 × 10^6^

**Table 8:** Mass spectrometric parameters for the detection of amphidinols (AM).

Ion-Source	
Capillary Voltage [kV]	2.00
Cone Voltage [kV]	45
Desolvation Temperature [°C]	600
**Gas Flow**	
Desolvation [L/h]	1000
Cone [L/h]	150
Nebulizer [bar]	7.0

## Supplementary Materials

The following are available online at https://www.mdpi.com/1660-3397/18/10/497/s1, Figure S1–S11: CID-spectra and fragmentation pattern of detected AM.
