# Supplementary material for: LC-MS/MS Method Development for the Discovery and Identification of Amphidinols Produced by Amphidinium
Source: Mar Drugs. 2020 Sep 29;18(10):497. doi: 10.3390/md18100497 (PMC7601433; doi:10.3390/md18100497)
Supplement: Supplementary file 1 [file marinedrugs-18-00497-s001.pdf]

marine drugs

Supplementary materials:

LC-MS/MS method development for the discovery and identification of amphidinols produced by *Amphidinium*

Marvin Wellkamp [1], Francisco García-Camacho [2], Lorena M. Durán-Riveroll [3], Jan Tebben [4], Urban Tillmann [4], and Bernd Krock [4,*]

1 Alfred-Wegener-Institut für Polar und Meeresforschung, 27570 Bremerhaven, Germany; marvin.wellkamp@web.de
2 Chemical Engineering Department, University of Almería, 04120 La Cañada, Almería; fgarcia@ual.es
3 CONACyT-Departamento de Biotecnología Marina, Centro de Investigación Científica y de Educación Superior de Ensenada, 22860 Ensenada, B.C. México; lduran@conacyt.mx
4 Section Ecological Chemistry, Alfred Wegener Institute, Helmholtz Centre for Polar and Marine Research, 27570 Bremerhaven, Germany; jan.tebben@awi.de (J.T.); urban.tillmann@awi.de (U.T.)
* Correspondence: Bernd.Krock@awi.de

Figure S1. HRMS-CID-spectrum and fragmentation pattern of LP-D $[M+Na]^+$ at *m/z* 1329.7548.
Figure S2. CID-spectrum and fragmentation pattern of AM-22 $[M+Na]^+$ at *m/z* 1667.9.
Figure S3. CID-spectrum and fragmentation pattern of LS-A $[M+Na]^+$ at *m/z* 1295.8.
Figure S4. CID-spectrum and fragmentation pattern of LP-B/LP-C $[M+Na]^+$ at *m/z* 1343.7, here shown on the structure of LP-B.
Figure S5. CID-spectrum and fragmentation pattern of putatively CAR-E $[M+Na]^+$ at *m/z* 1421.9.
Figure S6. CID-spectrum and fragmentation pattern of putatively AM-4 $[M+Na]^+$ at *m/z* 1323.8.
Figure S7. CID-spectrum and fragmentation pattern of AM-18 $[M+Na]^+$ at *m/z* 1381.8.
Figure S8. CID-spectrum and fragmentation pattern of AM-19 $[M+Na]^+$ at *m/z* 1483.8.
Figure S9. CID-spectrum and fragmentation pattern of AM-A $[M+Na]^+$ at *m/z* 1361.8.
Figure S10. CID-spectrum and fragmentation pattern of AM-A $[M+Na]^+$ at *m/z* 1463.8.
Figure S11. CID-spectrum and fragmentation pattern of LP-D $[M-H]^-$ at *m/z* 1305.8.

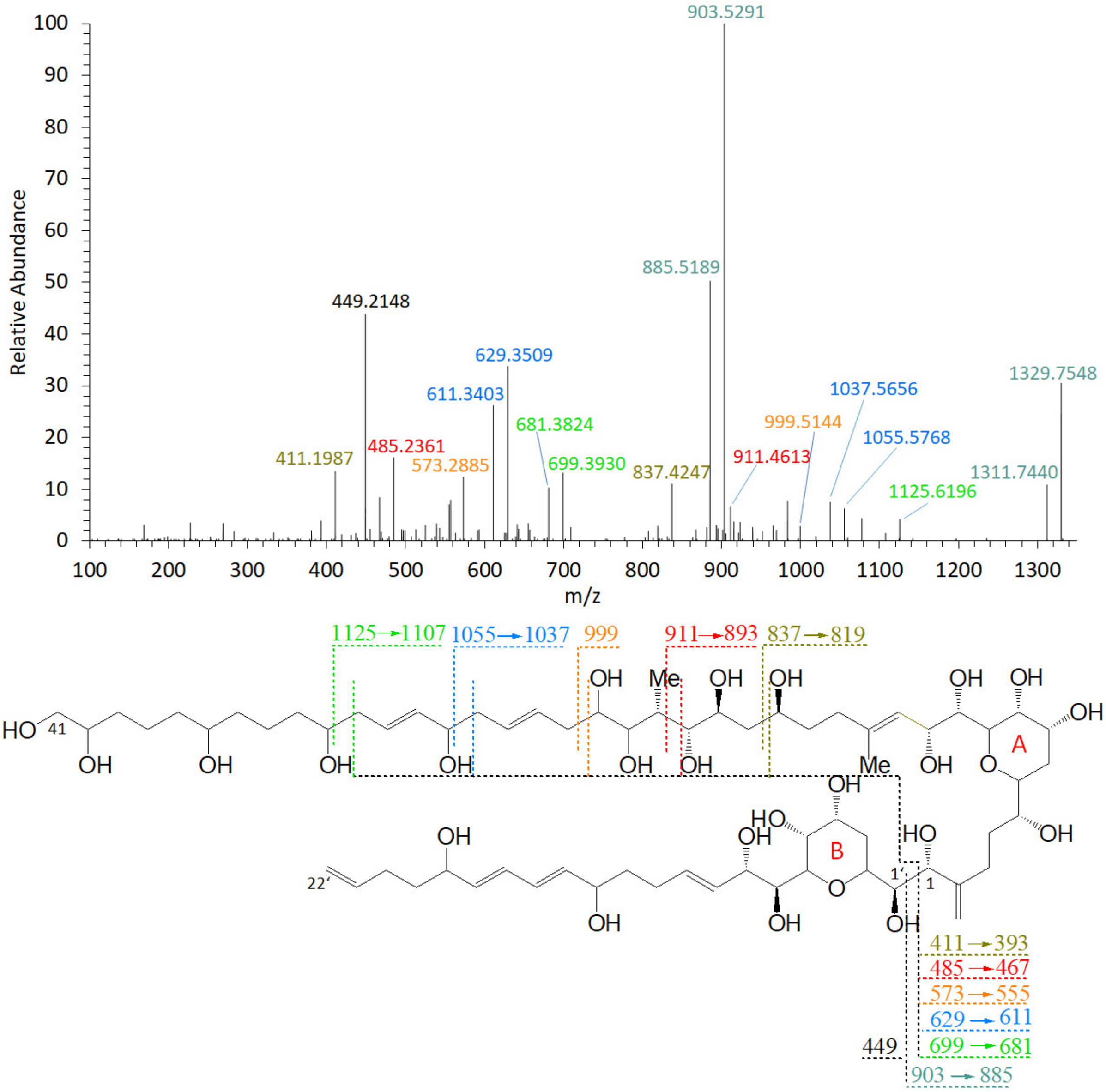

Figure S1. HRMS-CID-spectrum and fragmentation pattern of LP-D $[M+Na]^+$ at *m/z* 1329.7548.

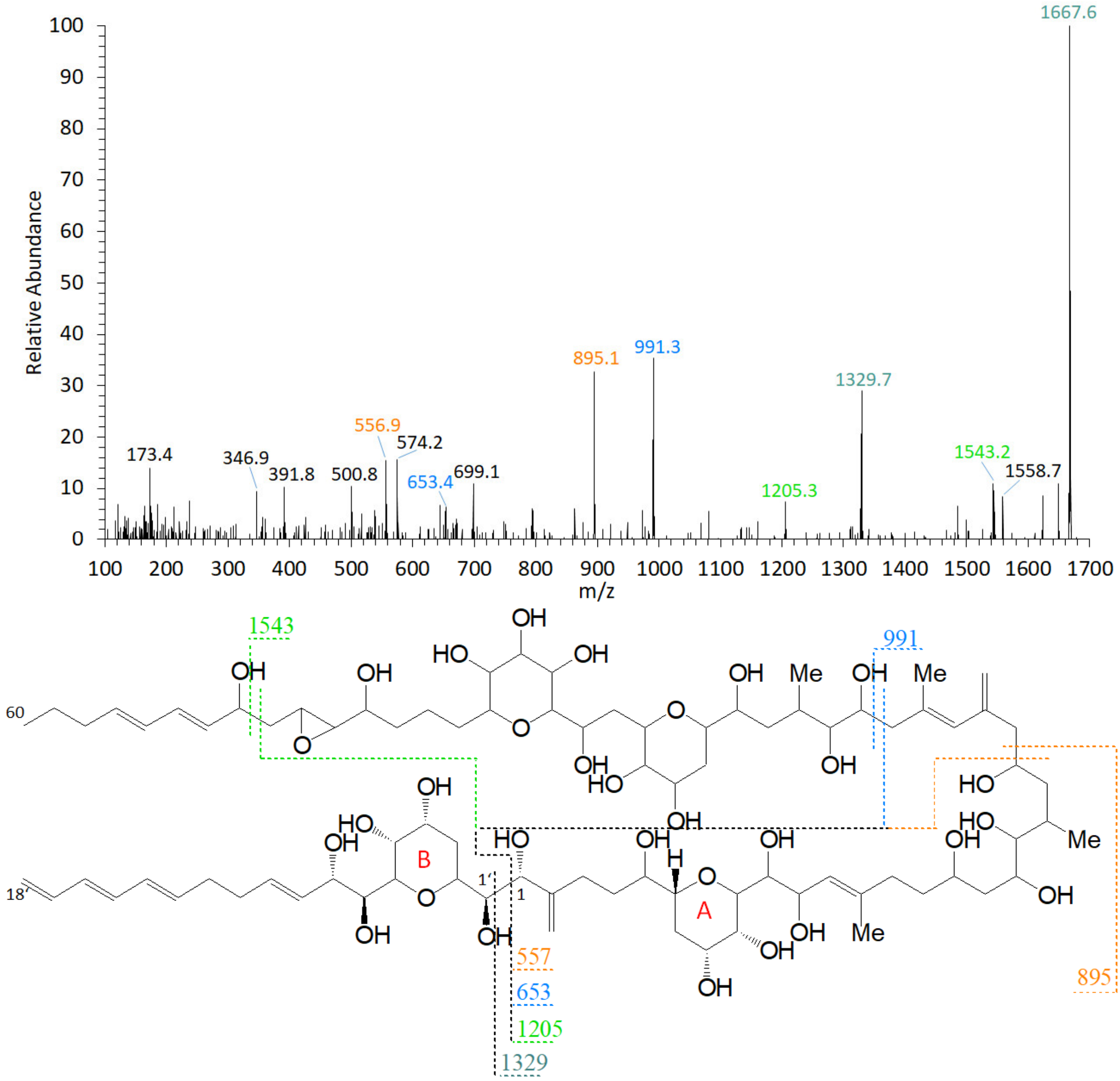

Figure S2. CID-spectrum and fragmentation pattern of AM-22 $[M+Na]^+$ at *m/z* 1667.9.

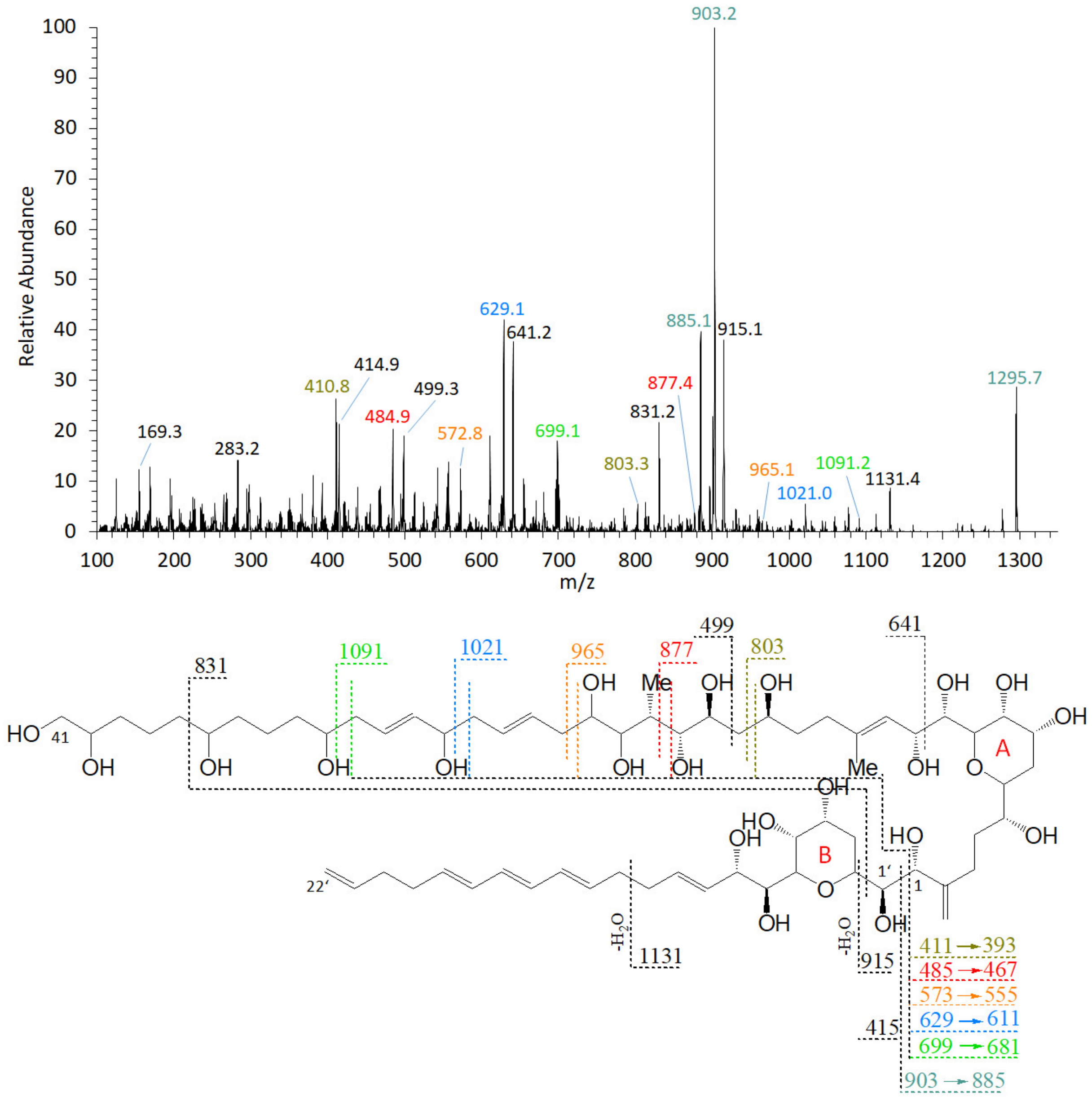

Figure S3. CID-spectrum and fragmentation pattern of LS-A [M+Na]$^+$ at *m*/*z* 1295.8.

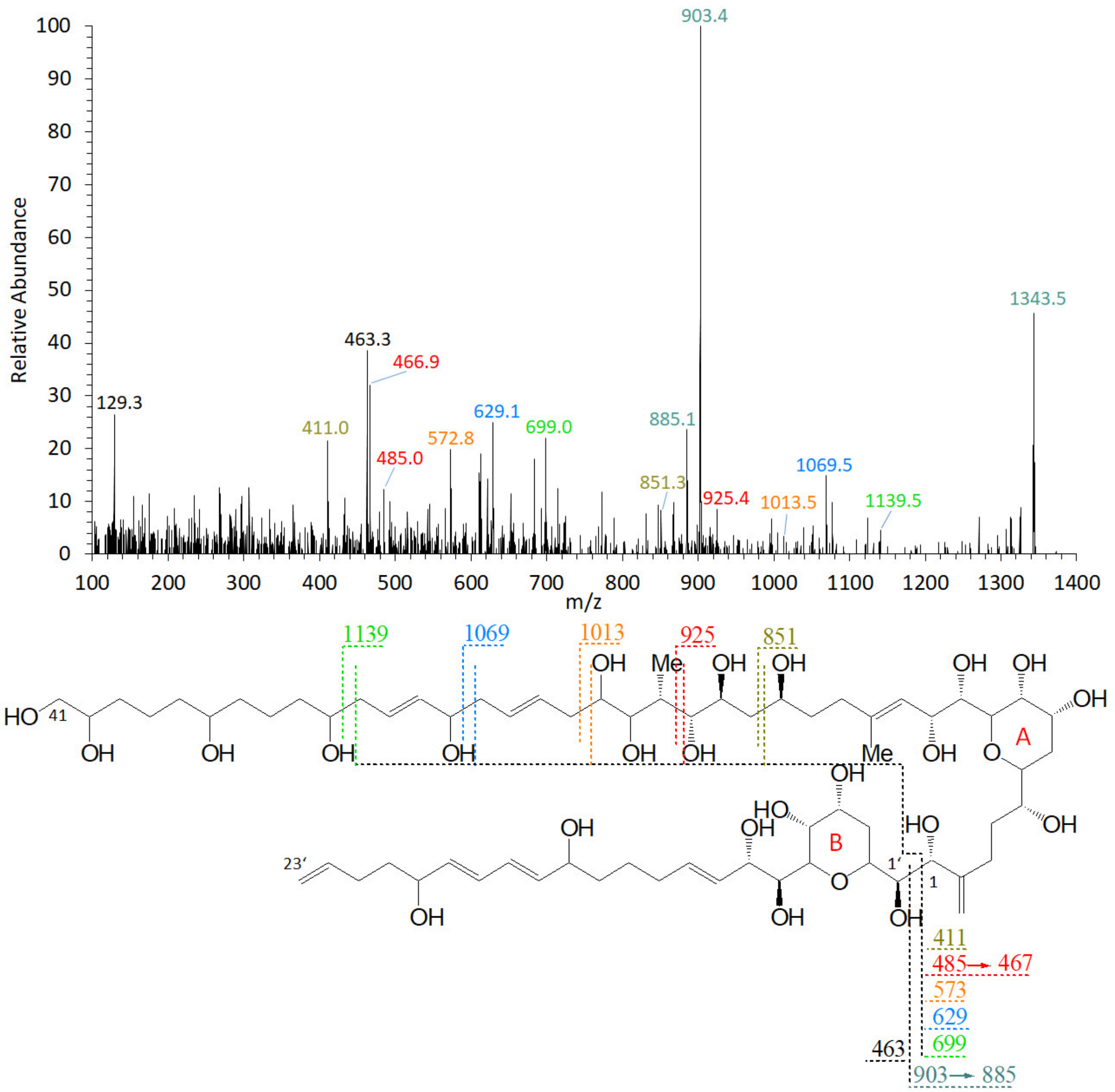

Figure S4. CID-spectrum and fragmentation pattern of LP-B/LP-C [M+Na]+ at *m*/*z* 1343.7, here shown on the structure of LP-B.

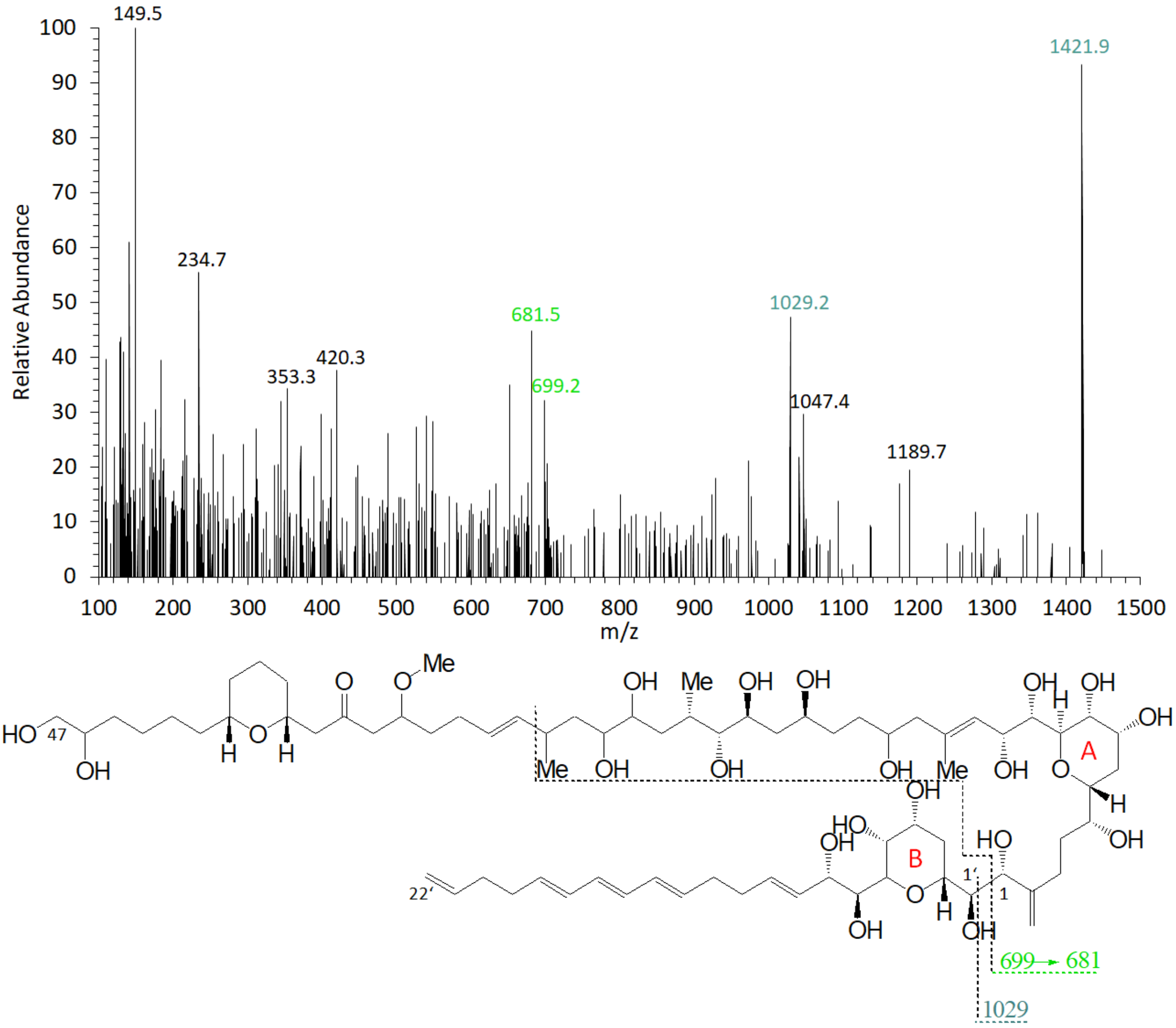

Figure S5. CID-spectrum and fragmentation pattern of putatively CAR-E [M+Na]+ at *m/z* 1421.9.

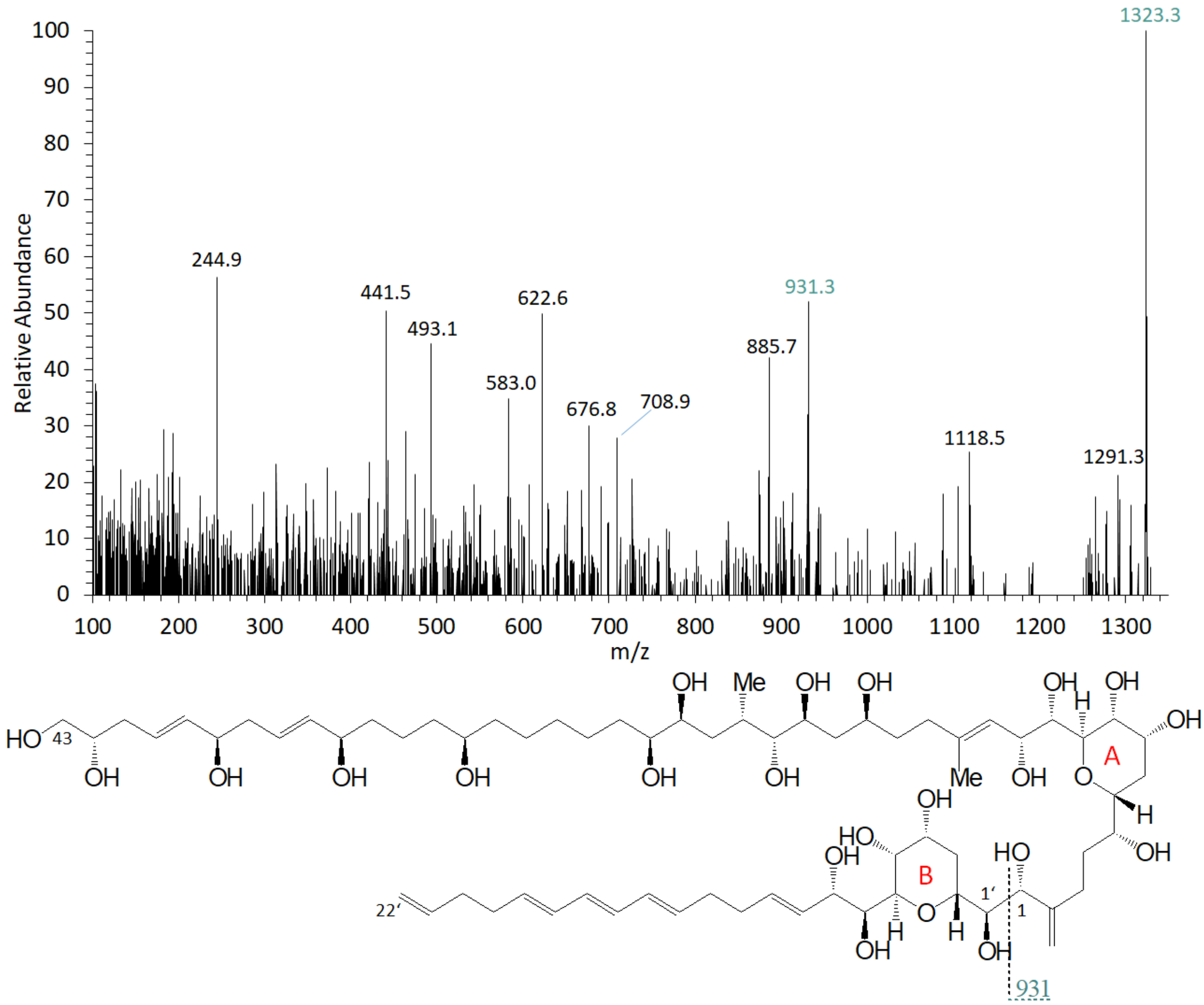

Figure S6. CID-spectrum and fragmentation pattern of putatively AM-4 [M+Na]+ at *m/z* 1323.8.

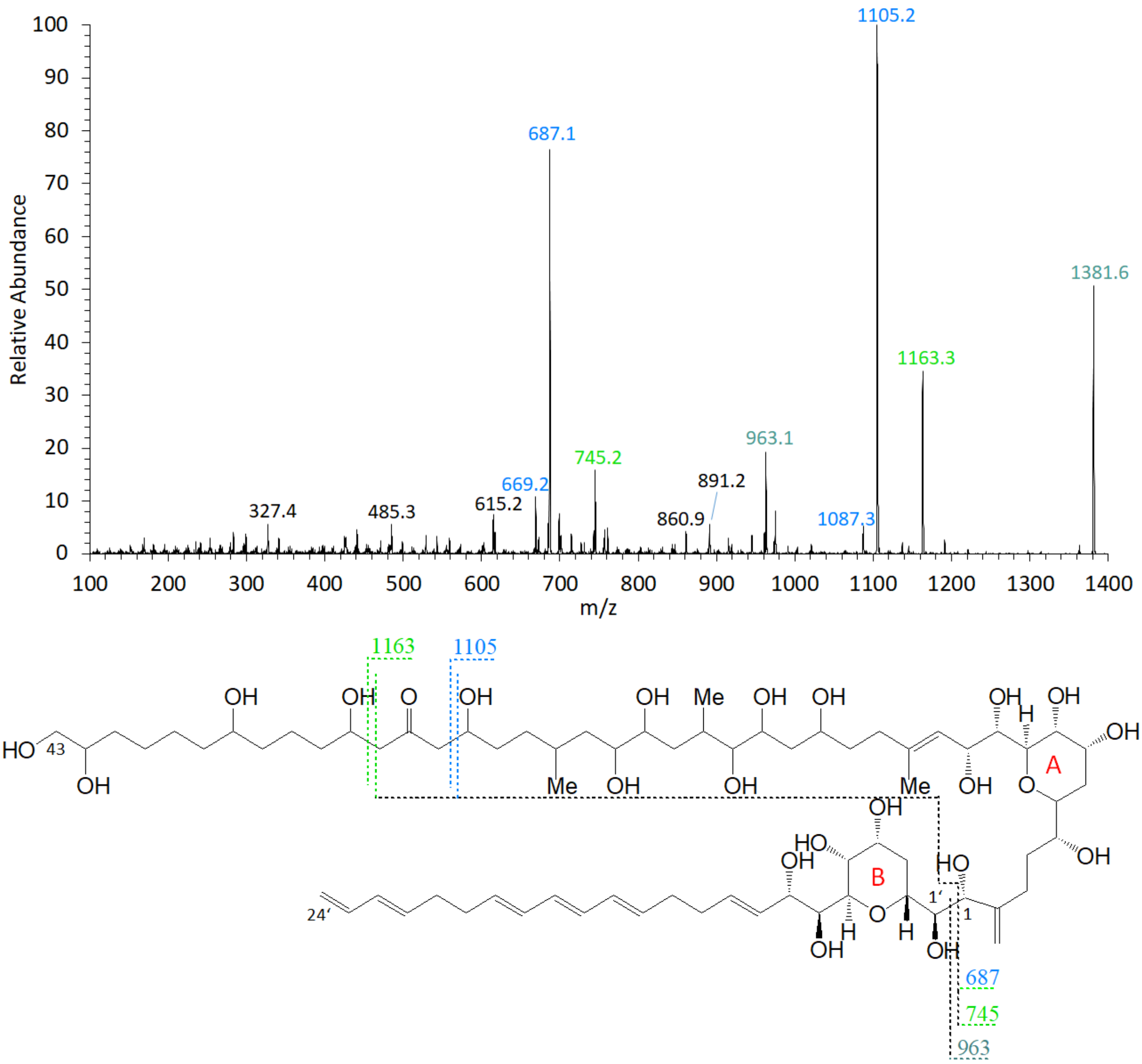
Relative Abundance
m/z
1105.2
687.1
1381.6
1163.3
963.1
745.2
669.2
891.2
615.2
327.4
485.3
860.9
1087.3
1163
1105
687
745
963
A
B
HO
OH
Me
24'
43
1'
1

Figure S7. CID-spectrum and fragmentation pattern of AM-18 [M+Na]+ at *m/z* 1381.8.

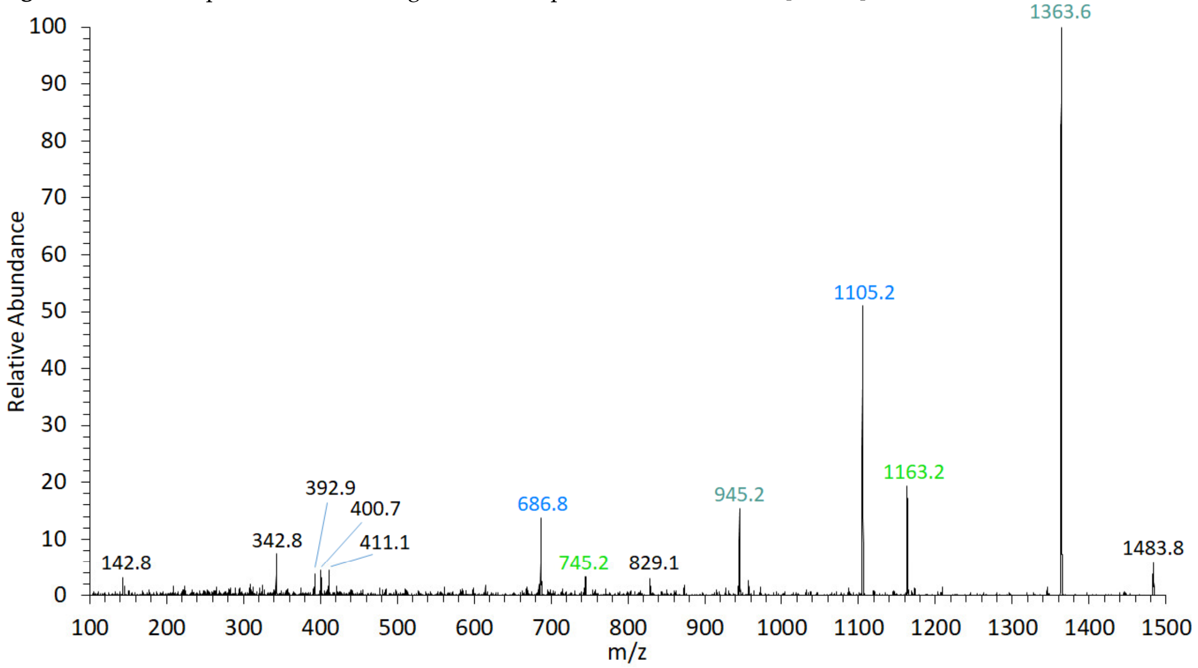

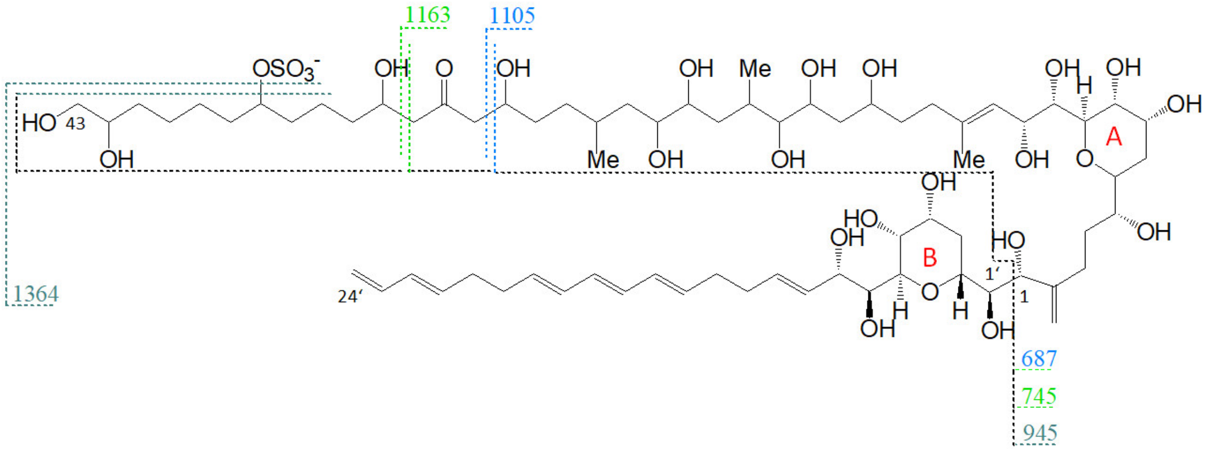

Figure S8. CID-spectrum and fragmentation pattern of AM-19 [M+Na]+ at *m/z* 1483.8.

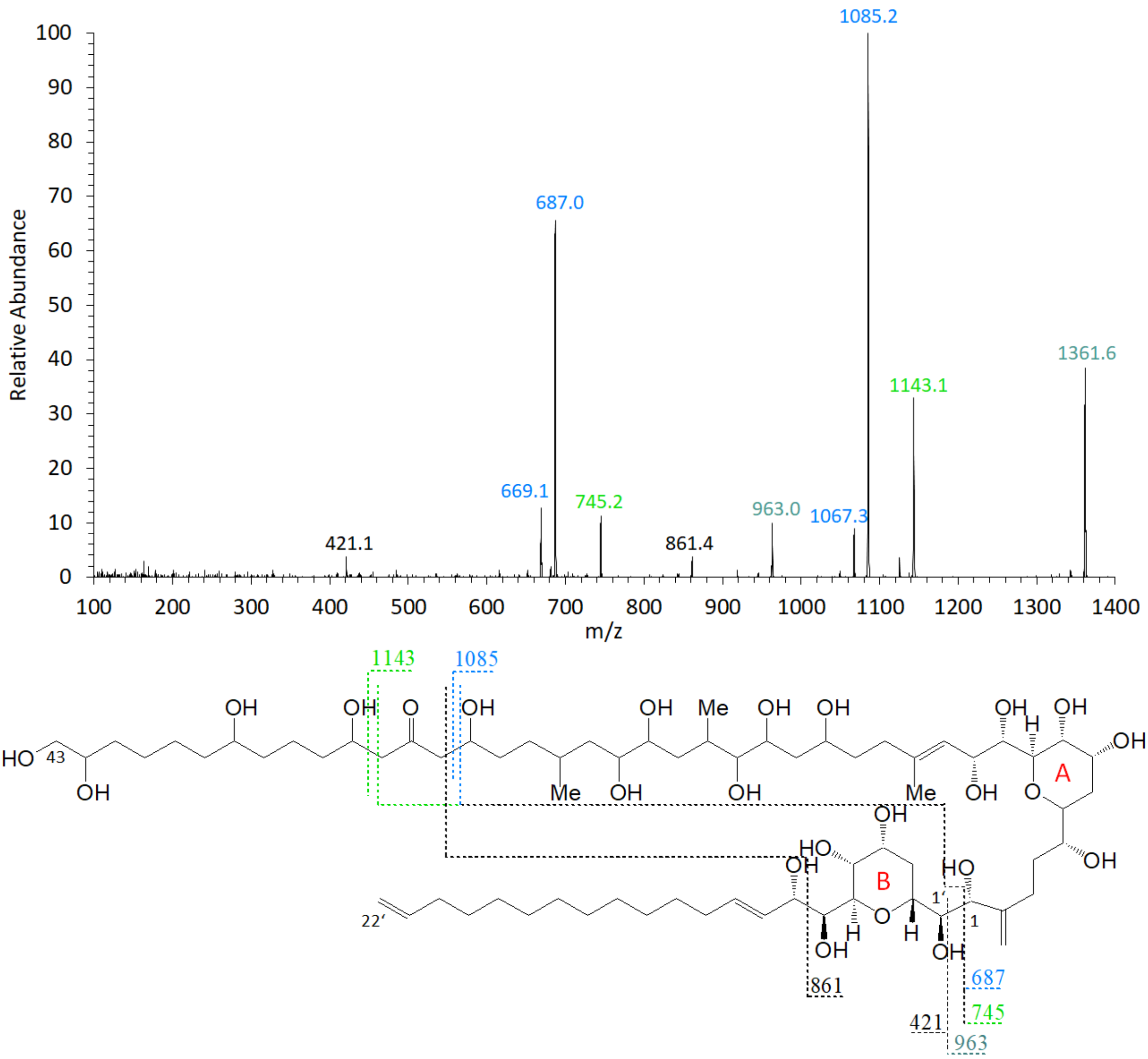

Figure S9. CID-spectrum and fragmentation pattern of AM-A [M+Na]⁺ at *m/z* 1361.8.

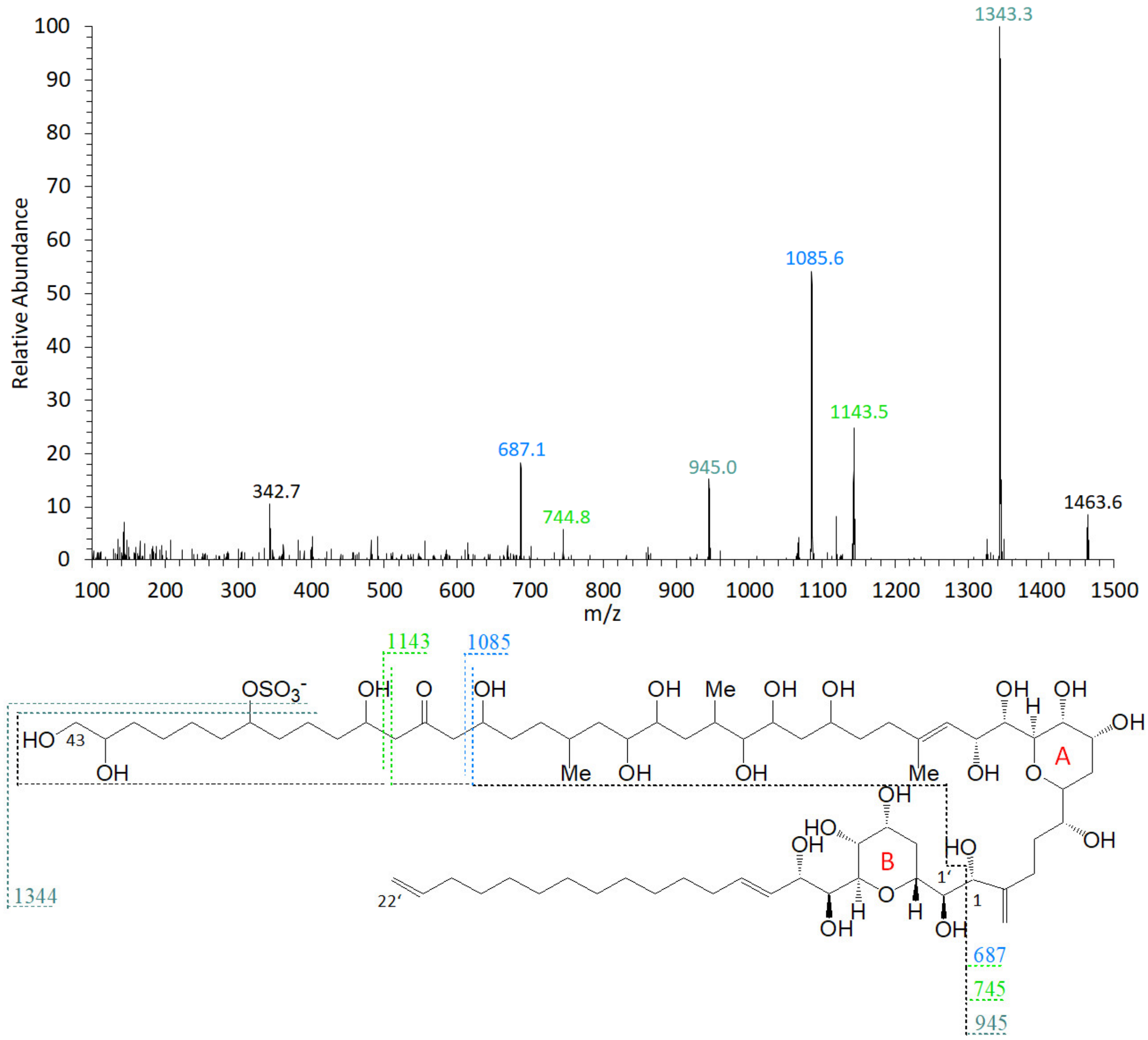

Figure S10. CID-spectrum and fragmentation pattern of AM-A $[M+Na]^+$ at *m/z* 1463.8.

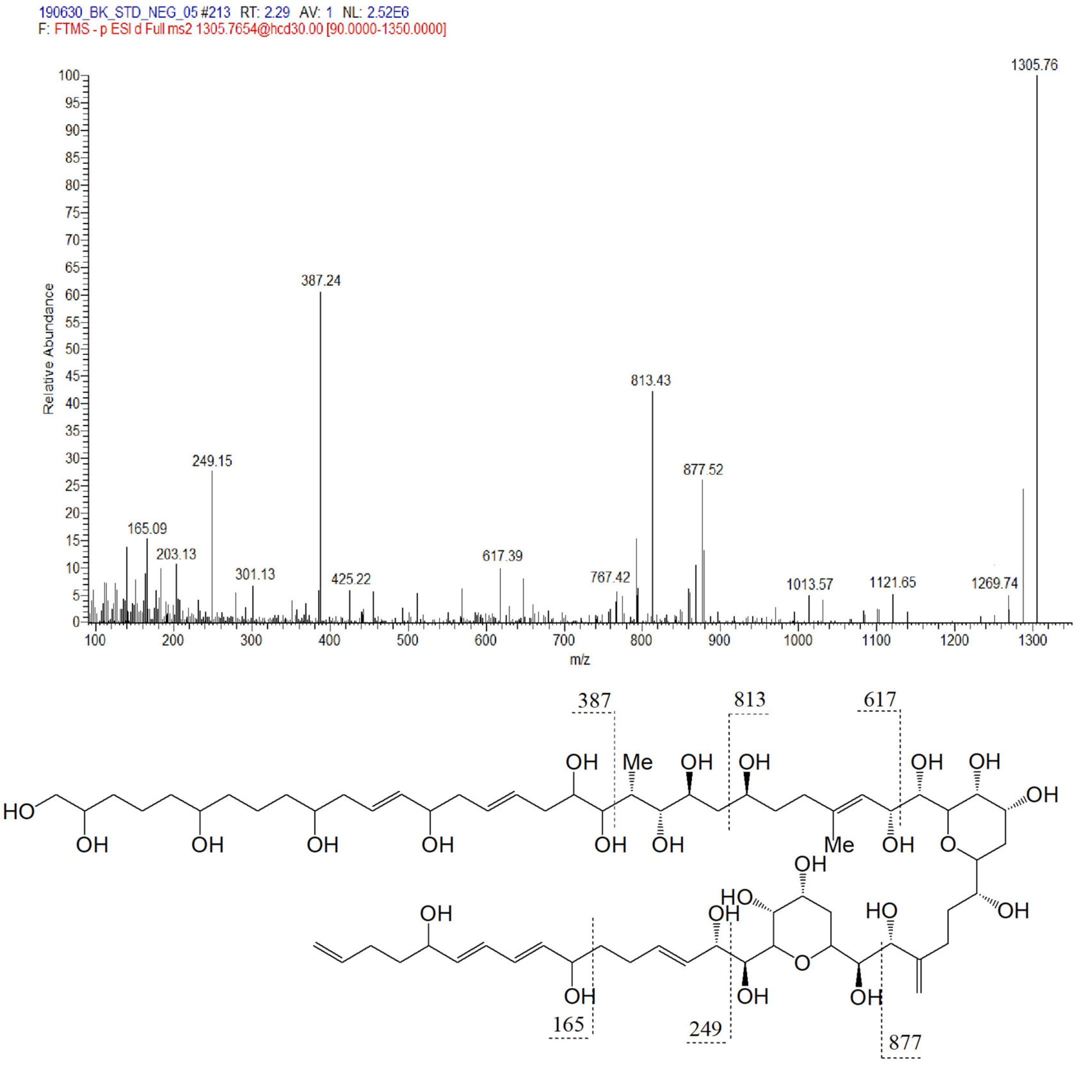

Figure S11. CID-spectrum and fragmentation pattern of LP-D [M-H]$^-$ at *m/z* 1305.8.

© 2020 by the authors. Licensee MDPI, Basel, Switzerland. This article is an open access article distributed under the terms and conditions of the Creative Commons Attribution (CC BY) license (http://creativecommons.org/licenses/by/4.0/).
